# Mechanomedicine in digestive surgery: a theranostic framework integrating mechanical diagnostics and therapeutic modulation across the perioperative continuum

**DOI:** 10.7150/thno.133908

**Published:** 2026-04-23

**Authors:** Ning Liu, Xiang Wang, Na Liu, Feng Xu, Ting Wen

**Affiliations:** 1 Department of Gastrointestinal Surgery, Hainan General Hospital, Hainan Affiliated Hospital of Hainan Medical University, Haikou 570311, Hainan, P.R. China.; 2 Department of Gastroenterology, Hainan General Hospital, Hainan Affiliated Hospital of Hainan Medical University, Haikou 570311, Hainan, P.R. China.; 3 Key Laboratory of Biomedical Information Engineering of Ministry of Education, School of Life Science and Technology, Xi'an Jiaotong University, Xi'an 710049, Shaanxi, P.R. China.; 4 Bioinspired Engineering and Biomechanics Center (BEBC), School of Life Science and Technology, Xi'an Jiaotong University, Xi'an 710049, Shaanxi, P.R. China.; 5 Hainan General Hospital, Hainan Affiliated Hospital of Hainan Medical University, Haikou 570311, Hainan, P.R. China.

**Keywords:** digestive surgery, anastomotic leakage, mechanical microenvironment, mechanical imaging, theranostics

## Abstract

Digestive surgery remains burdened by anastomotic leakage, postoperative obstruction, and highly variable functional recovery, even when operations are anatomically successful. Accumulating evidence indicates that these complications arise not only from morphology, but also from an unmeasured and unmodulated mechanical microenvironment, including tissue stiffness, anastomotic tension, perfusion-related shear stress, and intra-abdominal pressure. This review outlines a mechanomedicine framework for digestive surgery that treats these mechanical cues as actionable theranostic variables across the perioperative continuum, integrating mechanical diagnostics with targeted mechanical therapeutics. Multiscale biomechanics and mechanotransduction are first considered in relation to healing and fibrosis in the gastrointestinal tract. Preoperative mechanical profiling, using *in vivo* diagnostic techniques such as magnetic resonance elastography (MRE) and ultrasound shear-wave elastography (SWE), may refine surgical indications and support virtual surgical planning. Intraoperative mechanosensing tools, including functional lumen imaging, quantitative fluorescence perfusion, and portal pressure measurements, may provide real-time diagnostic thresholds to guide anastomotic site selection, reconstruction strategy, and resection extent. *In vitro* diagnostic platforms, including organ-on-chip models and mechanosensitive biosensors, have further clarified the mechanobiological basis of surgical complications and are informing the design of mechanoresponsive therapeutic systems. Postoperative mechanotherapy, encompassing continuous intra-abdominal pressure monitoring, mechanically tuned scaffolds, early mobilization, and mechanoresponsive drug-delivery systems, may contribute to early warning and personalized rehabilitation. Ethical and equity considerations are also relevant to this framework, including algorithmic bias, data governance, and equitable access across diverse healthcare settings. Together, these advances support a theranostic paradigm in which the same mechanical signals that diagnose risk may also help trigger, guide, or personalize therapy, pointing to a future in which digestive surgery is planned, performed, and followed up with more explicit and integrated control of the mechanical microenvironment to reduce complications and improve long-term function.

## 1. Introduction

Digestive surgery, encompassing a spectrum of procedures from routine colorectal resections to complex pancreaticoduodenectomies, serves as the cornerstone of curative treatment for a myriad of gastrointestinal malignancies and benign disorders 1-3. Over the past century, the field has witnessed a remarkable evolution in technical sophistication, transitioning from open laparotomy to minimally invasive laparoscopy and, more recently, to robotic-assisted platforms 4-6. These advancements have undoubtedly improved visualization and reduced surgical trauma. However, despite these technological leaps and the achievement of what is often described as “anatomical perfection” during reconstruction, the clinical community remains burdened by a persistent rate of severe postoperative complications, such as anastomotic leaks, postoperative bowel obstructions (ileus), and unpredictable recovery trajectories 7, 8. Anastomotic leak remains one of the most feared complications, and is associated with sepsis, reoperations, and even mortality 1-3. Conventionally, the etiology of these complications has been viewed through a morphological or biological lens, attributing failure to poor blood supply, bacterial contamination, or systemic comorbidities 9, 10. While valid, this perspective overlooks a fundamental physical reality: tissues are mechanical structures subject to physical forces 11-13. Accumulating evidence from the intersection of biomechanics and mechanobiology suggests that many of these complications arise not from anatomical errors, but from an unmeasured and unmodulated mechanical microenvironment such as excessive local tension, shear stress at the anastomosis, and tissue compliance mismatches 14, 15.

Historically, surgical planning and intraoperative decision-making have been guided almost entirely by morphology (visual inspection and static anatomy) and such an approach overlooks the dynamic mechanical microenvironment of healing tissues, which can be a decisive determinant of outcomes 16, 17. In short, surgeons have historically assessed “how things look” but not “how things feel”, and this could predispose to suboptimal results. For example, excessive anastomotic tension or shear forces can directly compromise healing, contributing to leak formation 11. Importantly, patients exhibit substantial inter-individual differences in tissue mechanical properties and responses, which a one-size-fits-all surgical approach fails to account for 18. Two patients may show similar anatomy on computed tomography (CT), yet differ markedly in tissue stiffness, fragility, or mechanical adaptability because of fibrosis, aging, or prior treatment. Such differences can explain why outcomes vary widely and why traditional risk models based purely on anatomy or basic vitals remain imperfect 19-21. By integrating patient-specific mechanical data (*e.g.*, measuring a patient's liver or bowel stiffness before surgery), surgeons can begin to personalize their strategies 22. Emerging studies suggest that using mechanics-guided planning, such as finite element modeling of anastomotic sites, can help tailor resection technique and tension management to the individual, thereby reducing complications. Mechanomedicine adds a functional, patient-specific dimension to precision surgery that morphology alone cannot provide 22, 23.

From a biophysical perspective, a surgical operation can be understood as a mechanical perturbation of tissues, after which the body enters a phase of force-driven healing and remodeling 24. Tissues are not static after surgery, as they respond to mechanical loads and gradually evolve toward a new equilibrium state as healing progresses 12, 13. In essence, wound healing in the gut is a dynamic biomechanical process: the surgical injury triggers a cascade where cells sense and respond to changes in mechanical cues in their microenvironment (*e.g.*, matrix stiffness, stretch, and pressure) over time 25-27. This process may also be interpreted within a non-equilibrium thermodynamic framework, in which surgery drives tissue away from its baseline state and healing gradually restores mechanical and structural stability 28, 29. Healing may be conceptualized as a process of progressive structural adaptation that reduces stress concentration and restores tissue continuity 28-30. In practical terms, this means that mechanical cues (*e.g.*, tension, pressure, and stiffness) continuously influence cellular behaviors during recovery, affecting scar formation and tissue function 11. When the mechanical microenvironment is favorable, healing is more likely to proceed appropriately; when excessive tension, abnormal shear stress, or inadequate mechanical support persists, the risk of dehiscence, fibrosis, or functional failure increases.

**Figure 1:**
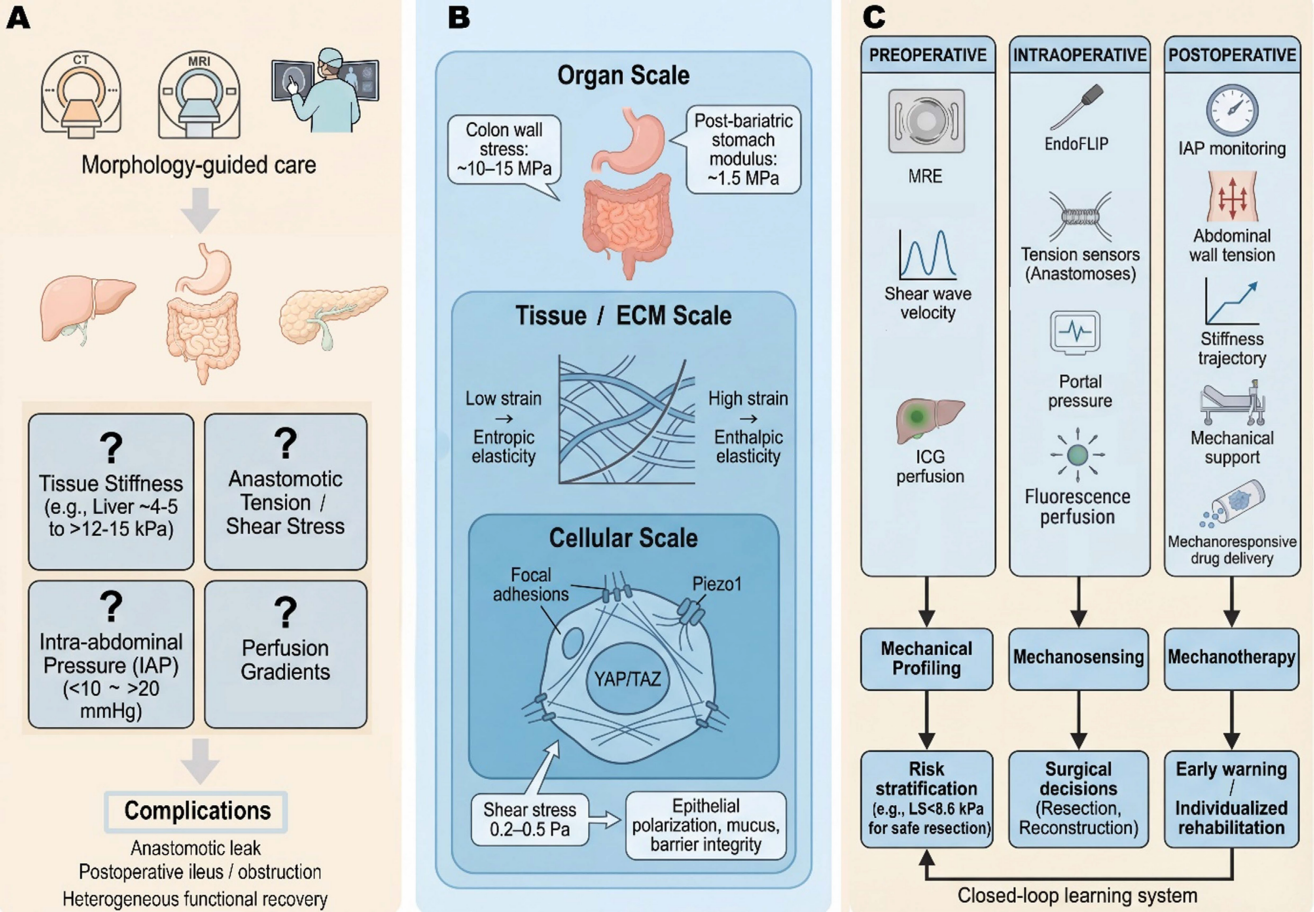
** Digestive surgery as a mechanically driven perturbation-healing process and the concept of mechanomedicine across the perioperative continuum. (A) Digestive surgery complications emerge from a mechanical “blind spot” in morphology-guided care.** Anastomotic leak, postoperative bowel obstruction/ileus and heterogeneous functional recovery remain common after digestive surgery, despite anatomically “perfect” operations 7, 8. Conventional planning relies primarily on morphology (*e.g*., computed tomography (CT), magnetic resonance imaging (MRI) and intraoperative visual inspection), without explicit quantification of key mechanical variables. In reality, these complications reflect unmeasured gradients in tissue stiffness, anastomotic tension, shear stress and intra-abdominal pressure, which vary widely between patients (*e.g.*, liver stiffness ranges from ~ 4-5 kPa in non-fibrotic to > 12-15 kPa in advanced fibrosis 18, 67, 68; intra-abdominal pressure (IAP) can rise from <10 mmHg to > 20 mmHg in abdominal compartment syndrome) 114. **(B) Multi-scale mechanical microenvironment in gastrointestinal tissues.** At the organ scale, digestive organs experience non-uniform stress and strain (*e.g*., colon wall tensile stress can reach ~ 10 - 15 MPa 11, 12; post-bariatric stomach modulus can drop to ~ 1.5 MPa as elasticity is altered) 36. At the tissue and extracellular matrix (ECM) scales, collagen and other fibers exhibit entropic elasticity at low strain and enthalpic elasticity at high strain, producing non-linear strain stiffening. Cells sense these changes via focal adhesion complexes, mechanosensitive ion channels (*e.g*., piezo type mechanosensitive ion channel component 1 (Piezo1)) and cytoskeletal tension, leading to nuclear translocation of Yes-associated protein/transcriptional coactivator with PDZ-binding motif (YAP (Yes-associated protein) / TAZ (transcriptional co-activator with PDZ-binding motif) and other mechano-effectors that reprogram gene expression 76, 79. In organ-on-chip systems, physiological shear stress in the range of ~ 0.2-0.5 Pa promotes epithelial polarization, mucus secretion and barrier integrity, illustrating how subtle mechanical cues modulate cell fate 49. **(C) Integrated mechanomedicine framework across the perioperative continuum.** In the proposed mechanomedicine paradigm, preoperative mechanical profiling (*e.g*., liver and pancreas stiffness by elastography, shear wave velocity, quantitative indocyanine green (ICG) perfusion) informs risk stratification and virtual surgical planning 19, 86; intraoperative mechanosensing (for example, functional lumen imaging for distensibility index, tension sensors on anastomoses, portal pressure measurements, fluorescence perfusion curves) guides on-table decisions about resection extent, anastomotic site and reconstructive strategy 23, 88; and postoperative mechanotherapy (*e.g*., intra-abdominal pressure (IAP) monitoring, abdominal wall tension tracking, stiffness trajectory of anastomoses, mechanical support and mechanoresponsive drug delivery) enables early warning and individualized rehabilitation 136. Longitudinal mechanical data feed back into subsequent preoperative assessment, creating a closed-loop learning system. The figure was created by Figdraw (www.figdraw.com).

The emerging field of mechanomedicine seeks to bridge this gap. Mechanomedicine is defined here as the clinical discipline that quantifies, interprets, and therapeutically modulates the mechanical microenvironment of living tissues to improve patient outcomes—distinct from mechanobiology (which studies how cells sense and respond to forces at the molecular and cellular level), biomechanics (which characterizes the physical properties and force distributions of biological structures), and bioengineering (which designs devices and materials for biological applications) 31-33. By treating mechanical cues not as passive bystanders but as actionable theranostic variables, surgical care may be fundamentally refined. This review presents a comprehensive, integrated theranostic framework for mechanomedicine in digestive surgery across the perioperative continuum, in which mechanical diagnostics and mechanical therapeutics are conceived as two inseparable arms of a unified strategy. The discussion begins with fundamental biomechanical principles, including solid stress, fluid shear, tissue stiffness, and mechanotransduction, and their roles in regulating biological responses. Three perioperative phases are then examined: preoperative mechanodiagnostics, intraoperative mechanical monitoring, and postoperative mechanotherapy. Each section summarizes recent advances demonstrating the clinical value of mechanics-based diagnostic and therapeutic approaches. These developments can be integrated into a unified theranostic paradigm and may inform surgical decision-making and patient care. This framework is intended to synthesize current knowledge in surgical mechanomedicine and identify opportunities for future investigation and clinical translation. Figure 1 illustrates this integrated mechanomedicine theranostic framework across the perioperative continuum and highlights digestive surgery as a mechanically driven perturbation-healing process.

## 2. The biomechanical basis of digestive system health and disease

To effectively modulate the mechanical microenvironment, it is essential to first understand the fundamental biophysical principles that govern the digestive tract 12, 34. Mechanomedicine is rooted in core biomechanical and mechanobiological concepts, such as solid stress, fluid shear stress, tissue stiffness, and mechanotransduction, that operate from the molecular scale to the whole organ level 11, 24, 35. The key terms are defined as follows: stress refers to the internal force per unit area within a material (units: Pa or kPa); strain is the fractional deformation of a material in response to stress (dimensionless); stiffness (or elastic modulus, *E*) is the resistance of a material to deformation under applied force (units: kPa or MPa); compliance is the inverse of stiffness, describing how readily a structure deforms under load; tension is the pulling force transmitted along a structure (units: N or mN); and shear stress (τ) is the tangential force per unit area exerted by a fluid flowing over a surface (units: Pa). These definitions are used consistently throughout the manuscript 11, 12, 24, 34, 35.

### 2.1 Solid mechanics: stress, strain, and the imperative of tension control

In digestive surgery, solid stress refers to the internal mechanical forces transmitted through the tissue matrix 36. These forces modulate tissue integrity and cellular behavior during both normal physiology and wound healing 14, 26, 37, and are particularly critical at the anastomotic site, where excessive tension is a primary driver of leakage 10, 23, 38.

The physics of anastomotic tension is governed by Laplace's law, which relates wall tension (*T*) in a cylindrical vessel to the intraluminal pressure (*P*) and radius (*r*) of the lumen:


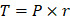
This relationship underscores the mechanical vulnerability of surgical anastomoses 11, 12, 36. Postoperative bowel distension or obstruction increases *r* or *P*, linearly amplifying suture-line tension 39-42. Healthy bowel typically withstands tensile stresses in the range of tens to hundreds of kPa, although local stress concentrations at suture or staple points may exceed the tissue's tolerance, causing micro-tears, ischemia, and dehiscence 2, 22, 23, 43-45.

This principle is particularly relevant in pancreatic surgery, where suture-parenchyma interaction is a primary determinant of postoperative pancreatic fistula (POPF) 1, 3, 46. Unlike the intestine's tough collagenous submucosa, the pancreas lacks a distinct capsule and is highly susceptible to solid stress 47. In a soft pancreas, low yield strength predisposes to suture cut-through under tension, leading to anastomotic failure 19, 46, 48.

Stress shielding—complete removal of mechanical stress—leads to tissue atrophy, analogous to Wolff's law in bone 13, 24. Gut tissues require physiological strain to maintain structural integrity 49-51; the surgical goal is therefore not to eliminate tension but to maintain it within an optimal range that stimulates healing without causing overstress 26, 40, 52, 53.

### 2.2 Fluid mechanics: the regulatory role of shear stress

Fluid shear stress (τ) is the frictional force exerted by a fluid flowing over a surface. In the digestive system, shear stress plays a significant role in vascular perfusion and luminal epithelium function, both of which are critical to healing after surgery 42, 49, 50.

Vascular shear stress and perfusion: Blood flow generates shear stress on endothelial cells that line the blood vessels 54, 55. Surgical interventions such as mesenteric torsion or the creation of stenotic anastomoses can induce complex, oscillatory shear stress patterns that influence endothelial function 54, 56, 57. Physiological shear stress promotes endothelial health by stimulating the production of vasodilators such as nitric oxide (NO), and it helps maintain an anti-thrombotic surface 37, 49. However, pathological shear stress—low-magnitude or oscillatory, often caused by low flow or anatomical kinking—triggers endothelial dysfunction, pro-inflammatory signaling (e.g., NF-κB), and a pro-thrombotic state, collectively compromising anastomotic perfusion 25, 58-60.

Luminal shear stress and barrier function: Shear stress from chyme flow in the intestinal lumen also regulates epithelial function 42, 50. *In vitro* organ-on-chip studies suggest that physiological shear stress in the range of approximately 0.2-0.5 Pa may promote mucus secretion and epithelial barrier formation, although the direct translation of these values to *in vivo* conditions remains to be established 37, 50. These microfluidic systems serve a dual role: they recapitulate the gut mechanobiological environment to diagnose the consequences of abnormal shear stress, and simultaneously serve as platforms for testing mechanoresponsive therapeutic interventions—exemplifying the *in vitro* theranostic approach.

### 2.3 Tissue stiffness and viscoelasticity: the material properties of healing

Tissue stiffness refers to a material's resistance to deformation under an applied force, and it is a key determinant of tissue integrity during healing 61. However, biological tissues, including those of the digestive tract, are not simple linear elastic materials but exhibit viscoelastic behavior, meaning they show both elastic (solid-like) and viscous (fluid-like) properties when subjected to deformation 34, 44, 62.

Non-linear strain stiffening: The extracellular matrix (ECM) of the gut, composed largely of collagen and elastin fibers, exhibits non-linear strain stiffening 34, 63. At low strain, these fibers are crimped and disordered, offering little resistance to stretching. As strain increases, the fibers straighten and the tissue becomes rapidly stiffer 44, 64. This is a protective mechanism that prevents over-distension. However, from a surgical perspective, this rapid stiffening can be problematic. As a suture is tightened, the tissue initially exhibits compliant behavior. However, with only a slight additional increase in tension, the tissue transitions into a high-stiffness regime, where stress rises nonlinearly. This abrupt increase in stiffness can elevate the risk of suture cut-through, particularly under certain loading conditions 43, 45.

Viscoelastic hysteresis: Tissues exhibit time-dependent behaviors—stress relaxation (decreasing stress at fixed length) and creep (gradual elongation under constant load)—that directly affect anastomotic stability 30, 38, 64. A suture line that feels secure at placement may loosen over hours due to these effects, compromising the seal 65, 66.

Pathological stiffness in disease: Fibrosis dramatically alters tissue stiffness. Cirrhotic livers exceed 12-15 kPa, altering handling characteristics and increasing fracture risk during suturing 21, 67, 68; fibrotic intestinal strictures similarly affect surgical handling and fibroblast behavior 56, 69. In the pancreas, stiffness is a critical biomarker: a soft pancreas (low stiffness) is the most significant risk factor for pancreatic fistula 19, 46, while chronic pancreatitis and pancreatic ductal adenocarcinoma produce a dense desmoplastic stroma with markedly elevated stiffness 70.

### 2.4 Mechanotransduction: the cellular interpreters of force

Mechanotransduction refers to the process by which cells convert mechanical forces into biochemical signals, enabling them to adapt to their mechanical environment 14, 58. This process plays a crucial role in wound healing, fibrosis, and cancer progression in the digestive tract 15, 18, 71.

***The integrin-cytoskeleton linkage:*** Integrins are transmembrane receptors that link the ECM to the cytoskeleton 15, 72. When cells encounter a stiff matrix, such as in fibrotic liver or scar tissue, integrins cluster and form focal adhesions, which recruit signaling proteins like Focal Adhesion Kinase (FAK) and Rho-associated kinase (ROCK) 72, 73. This activation increases cytoskeletal contractility, driving fibroblast differentiation into myofibroblasts, which are responsible for wound closure but also for pathological scarring and stricture formation 18, 71, 74. In the pancreas, this mechanism is driven by pancreatic stellate cells (PSCs). Upon exposure to a stiff mechanical environment or high fluid pressure, quiescent PSCs transform into an activated phenotype, synthesizing excessive ECM and perpetuating pancreatic fibrosis 47. Thus, a stiff mechanical environment promotes a cellular phenotype that exacerbates stiffness, creating a positive feedback loop of fibrosis 18, 75.

***YAP/TAZ signaling:*** YAP and TAZ translocate to the nucleus on stiff substrates or under high tension, acting as transcriptional co-activators that promote proliferation and cell survival 58, 76-78. Chronic activation by sustained mechanical stress drives fibrosis and cancer progression; notably, YAP-mediated suppression of the cGAS-STING pathway in macrophages dampens immune responses in both tumor progression and postoperative healing 14, 15.

***Mechanosensitive ion channels:*** Piezo1 and TRPV4 open in response to mechanical deformation, allowing calcium influx that triggers downstream intracellular signaling cascades 52, 79, 80. In the gut epithelium, activation of Piezo1 by physiological shear stress is essential for maintaining barrier integrity and promoting mucus secretion 49, 52. Conversely, excessive strain or high stiffness can lead to altered ion channel activity, triggering inflammatory responses in macrophages and potentially driving pathological tissue responses in the form of fibrosis or dysregulated healing 52, 81, 82.

**Figure 2:**
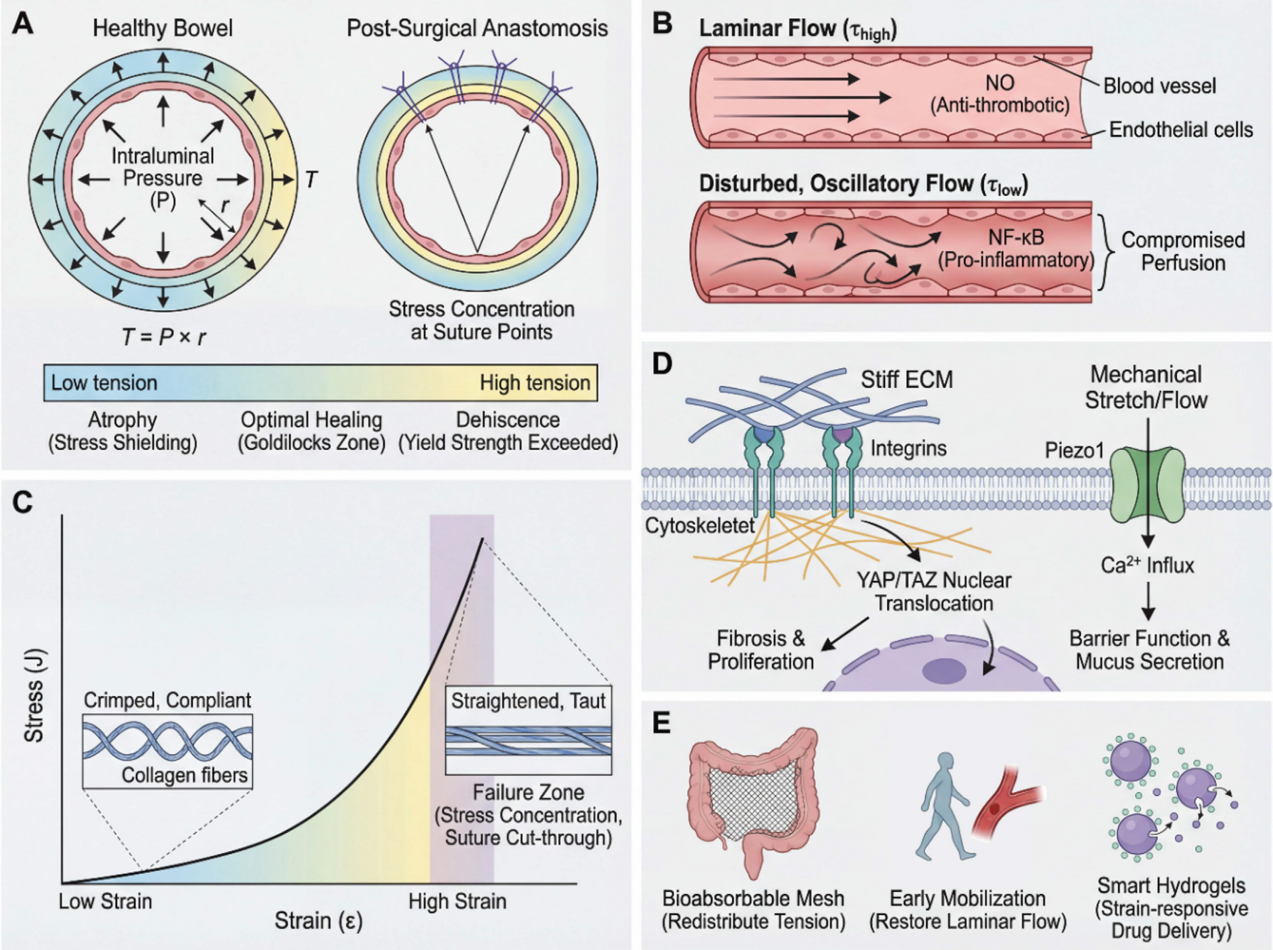
** Multiscale biomechanical mechanisms in gastrointestinal health and surgical disease. (A) Solid stress and anastomotic tension (macro-mechanical)**. The mechanical integrity of the bowel wall is governed by the Law of Laplace (*T = P × r*), where wall tension (*T*) is proportional to intraluminal pressure (*P*) and radius (*r*) 11, 12. In a healthy state, tension is distributed evenly. However, at the post-surgical anastomosis, increased pressure or dilation concentrates stress at suture points 22, 23. Excessive tension exceeding the tissue's yield strength can lead to micro-tears, ischemia, and dehiscence 10, 43, whereas stress shielding may cause atrophy; optimal healing occurs within a mechanical “Goldilocks zone.” **(B) Fluid shear stress and vascular perfusion (hemodynamic)**. Fluid mechanics play a regulatory role through shear stress (τ). Top: Laminar blood flow generates physiological shear stress, stimulating endothelial cells to produce nitric oxide (NO), maintaining a healthy, anti-thrombotic phenotype 49, 50. Bottom: Pathological conditions (*e.g*., torsion or stenosis) cause disturbed, oscillatory flow. This low-magnitude shear activates pro-inflammatory pathways (*e.g*., NF-κB), leading to endothelial dysfunction and compromised perfusion at the healing site 41, 109, 110. **(C) Tissue stiffness and non-linear strain stiffening (material properties)**. Gastrointestinal tissues exhibit non-linear elasticity. At low strain, collagen fibers are crimped and compliant (bottom inset). As strain increases, fibers straighten and become taut (top inset), causing a rapid, exponential increase in stiffness (J-shaped curve) 34, 102. Surgical tightening beyond this transition point risks entering the high-stiffness failure zone, where stress concentration leads to suture cut-through. **(D) Mechanotransduction and cellular response (cellular interpreter)**. Cells convert mechanical cues into biochemical signals via two primary pathways. Path 1 (Stiffness Sensing): Integrins link the stiff extracellular matrix (ECM) to the cytoskeleton, triggering the nuclear translocation of YAP/TAZ, which promotes gene expression associated with fibrosis and proliferation 72, 77. Path 2 (Stretch/Flow Sensing): Mechanical stretch or fluid flow activates mechanosensitive ion channels (*e.g*., piezo type mechanosensitive ion channel component 1 (Piezo1)), allowing Ca^2+^ influx to regulate barrier function and mucus secretion 25, 79. **(E) Clinical implications and mechanotherapy (translational solutions).** Application of biomechanical principles to “Mechanomedicine.” Vignette 1: Structural modulation using bioabsorbable meshes to redistribute tension and prevent stress concentration 118, 135. Vignette 2: Physiological modulation via early mobilization to restore laminar flow and perfusion 136, 138. Vignette 3: Smart materials, such as strain-responsive hydrogels, that adapt to the mechanical environment for targeted drug delivery 129, 171. The figure was created by Figdraw (www.figdraw.com).

In summary, the biomechanical principles reviewed here—solid stress, fluid shear, tissue stiffness, and mechanotransduction—are not merely theoretical constructs but have the potential to serve as clinically relevant biomarkers and therapeutic targets: the same mechanical signals that indicate pathological states also serve as targets for intervention 35, 83, 84. Incorporating these principles into routine clinical practice, as both diagnostic biomarkers and therapeutic targets, is the foundation of mechanomedicine across the perioperative continuum 4, 85. Figure 2 summarizes these multiscale mechanisms and their clinical implications.

## 3. Preoperative phase: mechanics-guided precision planning

Before the surgeon ever makes an incision, mechanomedicine can contribute valuable diagnostic information to risk stratification and surgical planning. Two major components are addressed: (1) mechanics-based diagnostics, encompassing both *in vivo* imaging modalities and *in vitro* diagnostic platforms, to assess tissue pathology in mechanical terms (e.g., stiffness, compliance), thereby refining surgical indications and strategies; and (2) mechanics-informed optimization of the surgical plan, using computational models or machine learning to simulate surgical scenarios and predict outcomes. This preoperative diagnostic phase is the first arm of the theranostic cycle: mechanical information gathered here directly informs and personalizes the therapeutic strategy.

### 3.1 Mechanical diagnostics for disease staging and surgical decision-making

MRE and ultrasound shear wave elastography (SWE) are well established in selected clinical contexts, particularly in hepatology, but their application in broader digestive surgical decision-making remains under active investigation 86, 87. Unlike CT/MRI, which depict anatomical structure, elastography provides a functional map of tissue mechanical properties. This has important implications for disease staging and theranostic decision-making, since many pathological processes—fibrosis, inflammation, tumors—alter tissue stiffness before morphological changes become apparent 20, 21, 61, 67, 68. These *in vivo* diagnostic readouts do not merely characterize disease; they directly guide therapeutic decisions, exemplifying the theranostic principle of using the same measurement to both diagnose and direct treatment.

One area with immediate clinical impact is chronic liver disease and surgical candidacy. For patients considered for liver resection (for tumor or other lesions), underlying liver fibrosis significantly affects the risk of postoperative liver failure. MRE and SWE can measure liver stiffness (LS) across the liver noninvasively 21, 68, 71. Recent studies have defined stiffness thresholds that correlate with fibrosis stage and surgical outcomes. For example, in patients with hepatocellular carcinoma (HCC), using two-dimensional shear wave elastography (2D-SWE), LS below about 8-9 kPa has been identified as a risk-stratifying threshold in selected cohorts (minimal fibrosis), whereas LS above ~ 12-15 kPa suggests advanced fibrosis/cirrhosis where resection plans should be more cautious 21. In one cohort, an LS < 8.6 kPa predicted low risk of post-hepatectomy complications (sensitivity ~ 89%, specificity ~ 74%), whereas patients with LS > 12 kPa had markedly higher complication rates and often benefited from adjusted surgical strategy (*e.g.*, smaller resection or portal pressure management) 21, 88. In essence, preoperative liver stiffness can serve as an objective risk indicator, analogous to ejection fraction in cardiology, and may support decisions regarding whether resection is likely to be tolerated safely. This represents a shift towards functional criteria for surgical indications, supplementing anatomical criteria like tumor size or location 21, 68, 71.

In pancreatic surgery, especially pancreatoduodenectomy, the texture of the pancreas is a well-known factor in outcomes. A soft, fatty pancreas is more prone to pancreaticojejunostomy leak (postoperative pancreatic fistula, POPF) than a firm, fibrotic pancreas 1, 19, 47. Now, tools like endoscopic transient elastography and SWE allow quantification of pancreatic stiffness preoperatively as *in vivo* diagnostic biomarkers. A lower shear-wave velocity (SWV) or elasticity indicates a softer gland. A recent study confirmed that SWV ~ 1.3 m/s in the pancreas (a very low stiffness) was associated with significantly higher rates of clinically relevant POPF 19, 47. In practical terms, if elastography reveals a very soft pancreas, the surgical team might need additional safeguards, for example, using a different anastomotic technique, placing a stent, or being prepared for rigorous drain management, to mitigate the anticipated risk of leak 19. This exemplifies the theranostic use of *in vivo* mechanical diagnostics: the elastographic diagnosis of a soft pancreas directly prescribes a modified therapeutic strategy. On the other end, elastography can help in tumor characterization: malignant pancreatic tumors tend to be substantially stiffer than benign lesions. MRE studies have shown that pancreatic cancers often exhibit stiffness > 3.5 kPa, whereas healthy pancreas is much softer 70. Multifrequency MRE (which probes tissue at different vibration frequencies to glean both stiffness and viscosity) has even been used to grade pancreatic neuroendocrine tumors, finding that higher-grade tumors have higher stiffness 70, 89. This kind of information could assist in borderline cases, for instance, if a pancreatic mass has intermediate imaging features, a high stiffness on elastography might push towards resection, whereas a low stiffness might suggest a benign or less aggressive lesion that could be observed 70, 89. While not yet standard of care and currently limited to specialized centers, these approaches are under active clinical investigation and hold promise for refining pancreatic surgical decision-making through integrated mechanodiagnostic-therapeutic protocols.

Another promising application is in colorectal liver metastases (CRLM). Beyond simply counting tumors and measuring their size, assessing the mechanical properties of metastases and the surrounding liver may predict how well a patient will respond to chemotherapy and how aggressive surgery should be 62, 90. A study used SWE to monitor liver metastasis stiffness during chemotherapy and found that a decrease in stiffness by > 13% after treatment was an excellent predictor of tumor response and longer progression-free survival (area under the curve (AUC) ~ 0.84 for response) 90. Essentially, if the metastasis softens with chemo, indicating tumor kill and less fibrotic stroma, the patient is likely responding well; if it remains stiff, the tumor might be chemoresistant. This could influence the timing of surgery, for instance, operating sooner on non-responders 90. MRE has also been explored for CRLM characterization, with some evidence that mechanical phenotyping of tumors correlates with their viability and invasive potential. Similarly, in hepatocellular carcinoma, tumor peripheral stiffness has been shown to modulate chemoresistance via mechanotransduction pathways, demonstrating that mechanical properties directly influence therapeutic outcomes 75. These are paradigmatic examples of theranostics in mechanomedicine: the same *in vivo* diagnostic measurement, namely tissue stiffness assessed by elastography, simultaneously characterizes disease state, predicts therapeutic response, and guides the selection and timing of treatment.

Even in scenarios like adhesive small-bowel obstruction (ASBO), mechanical diagnostics can help. Typically, when a patient has a partial bowel obstruction from adhesions, the decision to operate vs. continue conservative management can be tricky. One emerging tool is preoperative indocyanine green (ICG) fluorescence perfusion assessment, typically conducted during minimally invasive diagnostic laparoscopy 41, 60. ICG can be injected and its near-infrared fluorescence tracked to see how well the bowel wall is perfused beyond an obstruction. Quantitative ICG imaging, analyzing fluorescence intensity curves, allows estimation of whether a segment is ischemic or likely to recover 60. Recent reports indicate that delayed or weak ICG uptake in a distended bowel loop signals compromised perfusion and impending necrosis, meaning that surgical intervention is needed, whereas strong perfusion suggests the bowel might survive once decompressed 41, 60. Additionally, preoperative SWE of dilated bowel has been piloted to gauge bowel wall stiffness; a very stiff loop may indicate edema and fibrosis from prolonged obstruction, again nudging toward surgery, whereas a more compliant loop might resolve 91. These applications are still being refined, but they exemplify how mechanical and hemodynamic assessment is adding quantitative data to preoperative planning in emergency general surgery contexts.

Recent clinical studies suggest that mechanical readouts can influence surgical planning across stiffness, distensibility, and perfusion domains. In preoperative hepatobiliary patients, MR elastography-based liver stiffness alone identified severe fibrosis with an AUC of 0.85, whereas combining routine preoperative markers increased the AUC to 0.95, supporting the use of stiffness information to refine assessment of hepatic reserve before major resection 87. In foregut surgery, EndoFLIP-derived distensibility, which serves as a practical surrogate of luminal tension, showed that Hill fundoplication created a tighter gastroesophageal junction than Toupet fundoplication, with lower distensibility index (0.9 ± 0.4 vs 1.3 ± 0.6 mm²/mmHg) and compliance (25.9 ± 12.8 vs 35.4 ± 13.4 mm^3^/mmHg), illustrating how quantitative tension-related measurements can help calibrate reconstruction rather than relying on visual judgment alone 92. Likewise, in rectal cancer surgery, indocyanine green fluorescence angiography changed the initial surgical plan in 10.6% of patients undergoing low anterior resection, and no anastomotic leaks occurred in the subgroup in whom the proximal resection margin was modified, indicating that perfusion imaging can directly alter operative decision-making in real time 93. Together, these data support the concept that patient-specific mechanical information may complement anatomic imaging when tailoring operative extent, reconstruction, and perioperative strategy.

**Figure 3:**
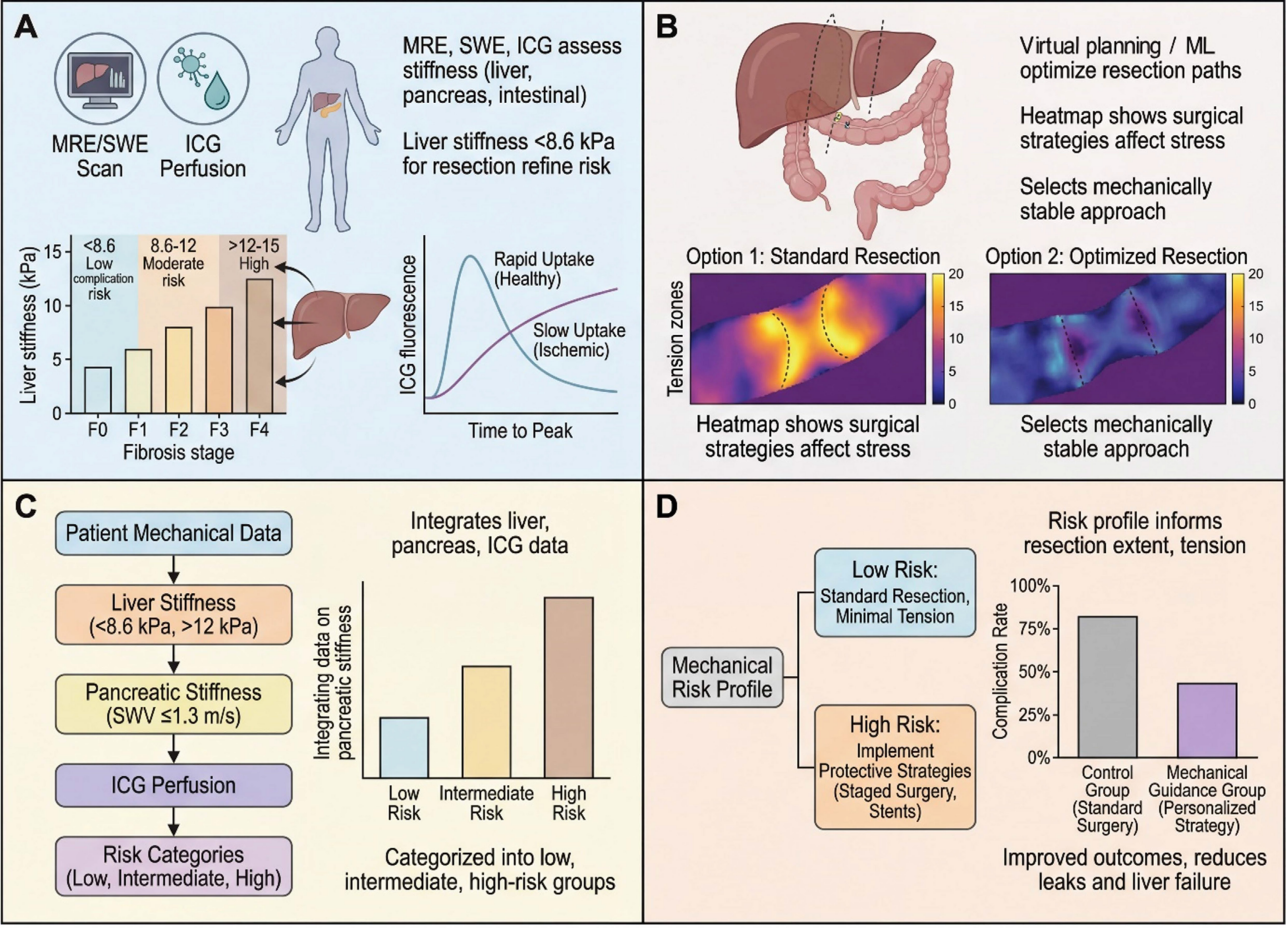
** Preoperative mechanodiagnostics and virtual stratification of surgical risk in digestive surgery. (A) Liver stiffness thresholds for safe hepatectomy.** Box-violin plots illustrate liver stiffness (LS) measured by magnetic resonance elastography (MRE) or shear-wave elastography (SWE) across fibrosis stages (F0-F1 to F4). Horizontal bands indicate risk-stratifying thresholds derived from recent prospective studies 67, 68, 86: LS < 8.6 kPa is associated with low rates of post-hepatectomy complications and liver failure, LS 8.6-12 kPa indicates intermediate risk, and LS > 12-15 kPa reflects advanced fibrosis/cirrhosis, in which resection extent should be carefully restricted or staged 87. Inset: complication rates increase stepwise across these strata, underscoring the value of stiffness-based selection. **(B) Pancreatic stiffness and risk of postoperative pancreatic fistula (POPF).** Preoperative shear-wave velocity (SWV) of the pancreatic parenchyma stratifies the risk of clinically relevant POPF after pancreatoduodenectomy 19. Patients with SWV ≤ 1.3 m/s (soft pancreas) exhibit the highest POPF rates, whereas risk declines with increasing stiffness 46. A receiver-operating characteristic (ROC) curve highlights the discriminative performance of a 1.3 m/s threshold (AUC ~ 0.75-0.80 in contemporary cohorts), providing a quantitative criterion for reinforcing reconstruction and drainage strategies 47. **(C) Mechanical phenotyping of colorectal liver metastases to predict chemotherapy response.** Elastographic monitoring of liver metastasis stiffness during systemic therapy reveals that a relative reduction in shear-wave velocity of ≥13% after initial cycles is strongly associated with radiologic response and improved progression-free survival, with an ROC area under the curve ~ 0.84 for this threshold 90. This demonstrates that tumor mechanophenotype is dynamic and prognostically informative 62. **(D) Intestinal perfusion and stiffness guide management of bowel obstruction.** Representative indocyanine green (ICG) fluorescence time-intensity curves show rapid, high-amplitude perfusion in viable bowel versus delayed, blunted uptake in strangulated segments 41, 60. In parallel, shear-wave elastography (SWE) can quantify intestinal stiffness, with values < 5 kPa suggestive of reversible obstruction and > 10 kPa raising concern for ischemia or chronic fibrosis 91. Together, these preoperative mechanodiagnostic tools support more precise selection of candidates for resection, the extent of resection and the timing of surgery across liver, pancreas and intestine. The figure was created by Figdraw (www.figdraw.com).

In summary, the preoperative phase now has tools to measure mechanical parameters (e.g., stiffness, tension, perfusion) both *in vivo* and *in vitro*, and leveraging this information can improve surgical decision-making. *In vivo* diagnostic tools, including MRE, SWE, and quantitative ICG perfusion imaging, provide patient-specific mechanical profiles that directly inform therapeutic planning. Complementarily, *in vitro* diagnostic platforms, such as organ-on-chip systems and mechanosensitive biosensors, have established the mechanobiological thresholds that underpin clinical decision criteria. Figure 3 illustrates some of these technologies and their impact on planning. For instance, by knowing a priori that a patient's liver is extremely stiff or that their pancreas is soft, the surgical team can stratify risk, perhaps opting for a two-stage liver surgery or preparing pancreatic stents and octreotide to mitigate fistula risk. This preoperative theranostic loop, in which mechanical diagnosis directly prescribes mechanical therapy, is the foundation of precision mechanomedicine. Critically, the mechanical data gathered preoperatively—liver stiffness thresholds, anastomotic tension predictions, and perfusion maps—directly prescribe the intraoperative strategy: which anastomotic site to select, how much tension to tolerate, and which real-time monitoring tools to deploy in the operating room.

### 3.2 Virtual surgical planning and simulation (mechanics-oriented optimization)

Beyond diagnostics, the preoperative period is increasingly benefiting from computational modeling and emerging artificial intelligence (AI) technologies to simulate surgeries *in silico*—approaches that remain largely translational but are advancing rapidly toward clinical integration. These methods allow surgeons to perform a virtual test run of different strategies on a patient-specific computational model, optimizing the plan and anticipating potential pitfalls; in the context of mechanomedicine, such simulations further incorporate patient-specific mechanical properties to predict how tissues will behave under surgical manipulation 63, 94-96.

Finite element modeling has become a cornerstone of biomechanics, and now it has been tailored to surgical planning. In the last couple of years, there have been notable successes in creating patient-specific FE models of organs, for example, modeling how a colon anastomosis will stretch and where stress will concentrate once constructed 17, 22, 94. The typical workflow involves taking the patient's imaging (CT/MRI) to build a 3D geometry of the organ, assigning material properties (*e.g.*, using elastography data for stiffness) 97-99, and then simulating surgical maneuvers like resecting a segment or suturing an anastomosis 63. Machine learning, especially deep learning, is augmenting this by handling complex data integration and speeding up computations 95, 96. Machine learning-driven models can analyze patterns from prior surgical cases (*e.g.*, images, mechanical data, outcomes) to assist in predicting complications and guiding intraoperative decisions 84. One example combined CT scans, elastography maps, and clinical variables to train an AI that outputs risk probabilities for anastomotic leak. These models achieved areas under the receiver operating characteristic (ROC) curve in the range of 0.75 - 0.85 in test cohorts 95, 96. In other words, by inputting a new patient's CT, stiffness, and lab data, the model might say “this patient has an 80% predicted probability of leak with a standard procedure.” Armed with that, the team can modify the plan, perhaps opting for a diverting stoma or reinforcing the anastomosis before proceeding 95, 96.

One concrete application is optimal anastomosis site selection in colorectal surgery. In Crohn's disease requiring resection of a long inflamed bowel segment, selection of the optimal margins remains challenging. Traditionally, it is by gross appearance 100. A computational model that maps strain distribution along the bowel under peristaltic load could identify regions of abnormal stiffness, if included in the anastomosis, would create stress concentrations. The surgeon could adjust the resection line to avoid that region 61, 95. Virtual surgery systems have been tested where different anastomotic configurations are simulated, and the one with the lowest peak stress is recommended 22, 35, 94. Likewise, in liver surgery, models can estimate the post-resection liver remnant shear stress and venous pressure. If a virtual right hepatectomy shows dangerously high stresses in the remnant, which predicts congestion or failure, the plan can change to a smaller resection or staged approach 57, 101.

Another emerging use is “digital twins” for surgery: creating detailed patient-specific computational models that integrate individual anatomy and tissue mechanical properties 97, 98. Such models have already been successfully applied in digestive surgery to characterize patient-specific gastric biomechanics and predict outcomes of different bariatric procedures 36, 102. Recent advances in breast surgery have further proven the clinical feasibility of rapid patient-specific finite element simulation pipelines using preoperative imaging 97. Extending these validated methodologies to broader gastrointestinal applications is a natural and highly promising next step 94.

Crucially, these simulations are becoming more data-driven and validated. It is not just theoretical: comparative studies have shown that FE-predicted anastomotic tension correlates with intraoperative sensor measurements 38. As computing power and algorithm sophistication grow, real-time or near-real-time simulation is on the horizon 38, 94. Research has proposed an “intraoperative digital twin” that acts like a surgical GPS. It is continuously updated by sensor data during an operation, thereby offering real-time guidance to the surgeon 83, 103. Although intraoperative digital-twin systems are still experimental, simpler data-driven applications have already entered clinical use. Preoperative MRE, for instance, provides a noninvasive assessment of liver stiffness and has shown value in perioperative risk stratification, including predicting postoperative recurrence in hepatocellular carcinoma and liver-related outcomes in chronic liver disease 86, 104.

Finally, on the AI front, deep neural networks—still at the translational research stage—have been trained on large datasets of past surgeries to detect patterns a human might miss. Related studies used a deep learning model to predict anastomotic leak in colorectal surgery patients. The model incorporated variables such as anastomosis level, patient comorbidities, and notably, intraoperative tissue quality assessments, which were based on the surgeon's grading of tissue frailty 35, 105. The model achieved an AUC ~ 0.80 and was better than traditional risk scores 106. If such models were augmented with actual mechanical measurements, such as elastography or tension sensor data rather than subjective tissue-quality scores, predictive accuracy might improve further. These findings suggest that AI/machine learning can synthesize multifactorial inputs, including biomechanical variables, to generate clinically useful risk predictions 85.

In summary, the preoperative phase is no longer just about imaging anatomy and optimizing medical comorbidities; it is becoming about mechanical diagnostics and virtual optimization. By identifying mechanical risk factors, such as a stiff liver, a soft pancreas, and abnormal flow—and by rehearsing the operation *in silico*, surgeons can significantly de-risk the actual surgery. This is a paradigm shift toward mechanics-informed precision surgery: treating each patient's tissues as unique materials and each operation as an engineering challenge to be optimized. When executed well, this should translate into fewer surprises in the operating room and better outcomes after. The next sections will delve into how these plans and predictions are applied and adjusted during the operation and afterwards.

## 4. Intraoperative phase: real-time mechanosensing and adaptive surgical maneuvers

Once in the operating room, mechanomedicine principles come into play through real-time *in vivo* diagnostic monitoring of mechanical conditions and targeted interventions to control the mechanical microenvironment of the surgery. This is perhaps the most immediately impactful phase, as decisions made intraoperatively can prevent complications before they start. The evidence base supporting intraoperative mechanosensing tools spans a spectrum: ICG fluorescence perfusion imaging and EndoFLIP distensibility measurement are supported by prospective clinical studies and are increasingly adopted in routine practice; intraoperative shear-wave elastography and implantable suture tension sensors are supported primarily by observational studies and early-phase trials; and closed-loop mechanical control systems remain at the experimental or the proof-of-concept stage. Two key aspects are addressed: (1) real-time mechanical monitoring, measuring tissue tension, distensibility, and perfusion to guide intraoperative decisions; and (2) targeted mechanical interventions, employing specialized materials and techniques to optimize the mechanical microenvironment. The intraoperative phase thus represents the most direct embodiment of theranostics in surgery: diagnostic sensors and therapeutic actuators operate simultaneously and in real time, with each diagnostic readout immediately triggering or adjusting a therapeutic response.

### 4.1 Real-time mechanical monitoring for surgical decision-making

Surgeons have long made qualitative judgments in the operating room, like “this anastomosis feels a bit tight” or “the tissue is stiff here”. Now, technology is translating those impressions into quantitative *in vivo* diagnostic readouts that can be objectively acted upon 45, 53, 54. A suite of intraoperative biosensing and imaging tools has emerged to quantify mechanical parameters, forming the diagnostic arm of the intraoperative theranostic cycle.

***Basic mechanical metrics:*** It is useful to recall the definitions of the mechanical terms (*e.g.*, tension, stress, strain), as they apply to surgical tasks 12, 22, 23, 94. Tension (*T*) typically refers to a force applied along a structure (in *N*, newtons). For a suture line or an anastomosis under pressure, one can estimate tension via Laplace's law: *T* = *P* × *r*, where *P* is the internal pressure and *r* is the radius 12. Stress (*σ*) is force per unit area, for example, within the intestinal wall, whereas strain (*ε*) is the fractional change in length (*ΔL*/*L₀*) 22. The elastic modulus (*E*) is the slope of the stress-strain curve (*Δσ*/*Δε*) for small deformations, essentially a measure of stiffness 22. Finally, for hollow organs intraoperatively, surgeons use the distensibility index (DI), defined as cross-sectional area (of the lumen) divided by pressure, with units mm^2^/mmHg. DI is directly measured by devices like endoscopic functional lumen imaging probe (EndoFLIP) in real time 40, 53, 107. These metrics, once abstract, are now being displayed on monitors during surgery to inform decisions.

***Colorectal anastomoses (preventing leaks):*** A critical intraoperative decision is whether an anastomosis is secure enough or if it needs revision. Traditionally, surgeons perform a leak test by insufflating air or fluid and seeing if anything leaks out of the suture line 108. ICG fluorescence perfusion assessment, now widely adopted in colorectal and hepatobiliary surgery, has been evaluated in randomized trials. The AVOID trial (over 1000 patients) found that while routine ICG assessment did not significantly reduce overall leak rates in an unselected population, in certain high-risk cases it helped identify poorly perfused segments and change the surgical plan 109. Quantitative perfusion thresholds are clinically useful: an ICG delay-to-peak exceeding ~60 seconds, or insufficient fluorescence intensity at the colorectal stump, indicates compromised perfusion and may prompt trimming to healthier tissue or creation of a protective stoma 59. In fact, trials in esophagogastric surgery illustrate how real-time data can prevent errors: by using ICG to select a well-perfused anastomotic site, thereby avoiding hypoperfused zones that cause leaks, studies have achieved a significant drop in leak rates, for example, from approximately 14% to 6% 110.

Beyond perfusion, mechanical integrity testing is standard. Surgeons perform an intraoperative leak test (air leak test) by pressurizing the segment, not to be confused with destructive burst pressure (BP) testing used in experimental settings 43, 108. A critical threshold often used is ~ 25 mmHg: a secure anastomosis should hold this pressure without leaking. If leaks occur (*e.g*., bubbles seen under saline), reinforcing stitches are applied 108. While experimental studies note that suture material choice (silk vs. polyglactin) may have less impact on ultimate strength than surgical technique 45, standardized intraoperative leak testing is widely advocated to minimize postoperative failure 108. Novel implantable sensors are being developed to continuously monitor anastomotic tension postoperatively. One prototype is a thin-film sensor embedded in the suture line which wirelessly transmits data and alerts staff to dangerous tension spikes caused by distension or coughing, prompting immediate interventions. This capability blurs the intra- and post-operative phases, exemplifying mechanomedicine's continuity 38, 111, 112.

***Antireflux (fundoplication) surgery:*** Here, achieving the “just right” tightness of the wrap around the esophagus is critical, since too tight causes dysphagia, too loose causes reflux 40. The EndoFLIP, now in routine clinical use for antireflux and bariatric procedures, has become a game-changer 40, 53. It is a balloon catheter with impedance sensors that measure the diameter of the gastroesophageal junction in real time at a set volume, giving a readout of DI 40, 53, 107. Surgeons can now titrate their fundoplication during surgery based on EndoFLIP readings. For instance, studies reported that at a 40 mL balloon fill, a cross-sectional DI > ~ 3.5-3.6 mm^2^/mmHg correlates with minimal postoperative dysphagia, meaning the wrap is not too tight 53. However, if DI is much higher (> 6.0 mm^2^/mmHg at 40 mL), the wrap might be too loose, potentially risking hernia recurrence or persistent reflux. Related research suggests aiming for a final DI between ~ 2 and 4 mm^2^/mmHg. In practice, if EndoFLIP shows a DI of 1.0 mm^2^/mmHg at 30 mL, indicating excessive tightness, the surgeon may consider loosening the wrap. Conversely, if it shows 6.5 mm^2^/mmHg at 40 mL (very loose), they might add an extra stitch 40, 53. A study provided evidence that using FLIP to guide these adjustments reduced both dysphagia rates and wrap failures 40. This is a prime example of intraoperative biomechanical guidance; the surgery outcome can be optimized by hitting a numerical target rather than solely relying on feel. EndoFLIP is now being explored in other domains too, like assessing esophageal strictures or even pyloric tightness during gastric surgeries 40, 53, 107.

***Liver surgery (hemodynamics):*** The resection of major liver volumes presents the primary clinical challenge of preventing post-hepatectomy liver failure, specifically small-for-size syndrome, wherein the remnant liver is incapable of sustaining the portal inflow. Consequently, intraoperative measurement of portal venous pressure (PVP) following vascular occlusion or test-clamping has emerged as a critical decision-making guide 21, 68, 88. A post-resection PVP exceeding 20 mmHg is a known risk factor for liver failure 113. In such a case, one might abort a major resection or perform an additional procedure to modulate portal pressure, such as a splenectomy or the creation of a shunt 21, 88. In one recent study, patients with PVP above 20 had a significantly higher incidence of liver failure; keeping PVP < 18 was associated with safer outcomes. Some liver units have adopted an intraoperative algorithm: measure PVP after resection; if ≥ 20 mmHg, consider interventions or stage the resection 68. Quantifying a parameter like portal pressure, which replaces the subjective assessment of liver congestion with an objective threshold, is a proactive strategy analogous to how cardiothoracic surgeons guide therapy using pulmonary artery pressures 88.

***Pancreatic surgery (stiffness monitoring):*** Preoperative pancreas stiffness has been mentioned, and intraoperatively there is also interest in directly measuring pancreatic texture 19, 46, 47. Some specialized centers now perform intraoperative ultrasound elastography on the pancreas remnant after resection—a translational practice not yet widely standardized—to objectively quantify stiffness. A shear modulus can be calculated 47. For a nearly incompressible tissue like pancreas, E ≈ 3μ. If elastography confirms a very low stiffness, consistent with a soft gland, the surgeon might take additional precautions for the pancreatico-enteric reconstruction 19, 46. These may include using a duct stent, placing more drains, or in a high-risk case, even considering externalizing the pancreatic duct 47. Conversely, a stiff gland might allow a tighter anastomosis or fewer adjuncts. These elastography devices in the operating room are an extension of the preoperative concept, giving real-time feedback on the organ's mechanical state to tailor reconstruction 19, 46.

***Adhesiolysis and small bowel:*** When operating for adhesive obstruction, one risk is inadvertent enterotomy and another is postoperative re-adhesion. While mechanical monitoring is less established here, some interesting research has looked at intra-abdominal pressure and wall tension during these surgeries. Clinical studies suggest that keeping insufflation pressures as low as feasible during laparoscopy, for example around 8 mmHg in low-pressure protocols, may reduce the mechanical stress on tissues and improve postoperative recovery 114, 115. Some surgeons now practice “low-pressure” laparoscopy for bowel obstruction for this reason, in combination with careful fluid management to maintain perfusion. Doppler flow probes or ICG angiography can also be used intraoperatively to ensure the released bowel loops regain adequate perfusion. All these measures revolve around monitoring and controlling key mechanical factors, such as pressure, tension, and perfusion, to optimize outcomes during surgery 41, 60.

In summary, the intraoperative diagnostic toolkit is rapidly expanding. Mechanical data acquired during surgery, including anastomotic tension, perfusion indices, and distensibility measurements, may help define baseline parameters for subsequent postoperative monitoring and rehabilitation. Available intraoperative tools include fluorescence imaging for blood flow, probes for pressure and tension, imaging for stiffness and distensibility, and AI-enhanced laparoscopic video analysis that may indicate tissue properties. By quantifying critical mechanical parameters on the spot, surgeons can make informed therapeutic choices, revise the anastomotic site, place additional reinforcing sutures, and adjust insufflation. This real-time coupling of *in vivo* mechanical diagnosis with immediate therapeutic action is the operational definition of intraoperative theranostics. Table 1 provides an overview of the correspondence between key mechanical parameters, instrumentation, and clinical scenarios across the perioperative continuum, illustrating how each diagnostic modality is paired with a specific therapeutic response.

### 4.2 Targeted mechanical interventions during surgery

Mechanomedicine also encompasses intraoperative interventions, representing the therapeutic arm of the theranostic cycle, that actively shape the mechanical microenvironment on the basis of real-time diagnostic readouts 32.

Mechanical compatibility between biomaterials and native tissue is a key intraoperative principle: sutures, meshes, and scaffolds should be selected for mechanical properties compatible with native tissue 11, 48. Excessive suture stiffness may promote cut-through under tension, whereas insufficient elasticity may fail to maintain tissue approximation under load 65, 66. Next-generation sutures—combining natural polymers with graphene, or fabricated from poly(3-hydroxybutyrate-co-4-hydroxybutyrate) copolymers—achieve elasticity closer to soft tissue, allowing the suture to accommodate postoperative swelling while gradually transferring load to the healing tissue 48, 66, 116.

Emerging work indicates that intelligent robotic suturing may be enabled by both real-time visual perception and mechanically encoded force control: one study demonstrated real-time articulated-joint detection for robotic feedback 101, whereas another showed that slipknot-gauged sutures improved knotting-force precision, enhanced tissue perfusion in preclinical models, and allowed vision-based robotic stopping during suturing 117. Staple-line reinforcement with bovine pericardial strips or bioabsorbable glycolide felts—an established clinical practice in bariatric and thoracic surgery—distributes force more evenly along the staple line, reducing peak stress by over 30% and improving mechanical boundary conditions for healing 22, 94, 118.

Another intraoperative domain under experimental investigation is pharmacomechanical modulation: locally delivering drugs to influence the mechanobiology of healing 44, 81. When mechanical diagnosis identifies a high-risk anastomosis (e.g., soft pancreas by intraoperative elastography), targeted pharmacological agents are applied locally. For instance, researchers are investigating the use of matrix metalloproteinase (MMP) inhibitors or collagen crosslinkers at anastomosis sites to modulate the rate of extracellular matrix (ECM) degradation 119. Although a certain degree of MMP activity is required during early postoperative remodeling, excessive activity may weaken the anastomosis 71. Studies in other tissues, such as spinal cord, suggest MMP inhibition can modulate scarring 120, a concept potentially applicable to anastomosis. Another target is ROCK, part of the pathway that leads to myofibroblast contraction and fibrosis. Using a ROCK inhibitor locally can potentially reduce hyper-contraction of myofibroblasts and limit scar and stricture formation 56, 73. While these are not yet standard, the key point is that mechanotransduction pathways, such as YAP/TAZ and FAK-RhoA, can be pharmacologically modulated 37, 58, 78. For instance, anti-fibrotic gels containing agents such as halofuginone or losartan have been explored as local strategies to reduce adhesions and strictures by modulating TGF-β signaling and myofibroblast activation 58, 71, essentially trying to dial down the excessive mechanical signaling that leads to over-scarring 80.

A notable concept is the neuro-immuno-mechanical triad in wound healing 26: surgical dissection simultaneously induces mechanical injury, nerve disruption, and immune activation. Mechanical stress modulates immune cell behavior, while inflammatory responses in turn reshape tissue mechanics through edema and fibrotic changes 26, 37. Exemplifying mechanosensitivity, macrophages polarize into a pro-inflammatory M1 state under high stiffness or strain, conversely adopting a pro-healing M2 phenotype in soft environments, thereby guiding the inflammatory response 72, 78, 81. Thus, control of mechanical conditions may also modulate the immune response in a manner that favors healing 26. In adhesive small bowel obstruction surgery, for example, simply releasing tension by lysing adhesion bands, followed potentially by maintaining low-pressure peritoneal CO_2_ insufflation to prevent organs from recoalescing tightly, might reduce the drive for M1 macrophages and intense fibrosis 114, 121. Additionally, drugs that target mechanotransduction in immune cells are being explored, e.g., modulators of YAP/TAZ in macrophages to encourage a healing phenotype 58, 82, 122. These approaches remain experimental, but underscore a key insight: the mechanical microenvironment and immune response are intimately linked 123. Simple intraoperative measures—minimizing unnecessary traction, keeping tissues moist, and employing gentle handling—are low-tech ways to favor a pro-healing milieu 74.

Local pro-healing therapies represent a further intraoperative strategy. Small trials and animal studies show that fibrin sealant mixed with bFGF improves microvessel density and anastomotic burst strength 11, 58, 124, 125. A key challenge is controlled release: unregulated growth factor delivery risks leaky vessels or exuberant granulation 77, 126. Biodegradable 3D/4D-printed scaffolds—currently at prototype and the proof-of-concept stage—address this by embedding growth factors or anti-fibrotics for timed release tuned to the healing timeline, with prototype smart anastomotic patches providing initial mechanical support before dissolving as tissue regains strength 118, 127, 128.

Direct mechanical stimulation represents an experimental intraoperative strategy, currently supported only by animal model data 129. In animal models, low-frequency vibration (~50 Hz, 5 minutes) applied to a completed intestinal anastomosis improved collagen alignment and smooth muscle actin expression without tissue damage 129. Periodic tension cycling during surgery may similarly condition tissue to handle postoperative strains 25, 79, 130. These approaches remain preclinical but are grounded in mechanotransduction: controlled mechanical stress activates pro-healing pathways, whereas either excessive or absent stress is detrimental 52, 72, 74, 78.

**Figure 4:**
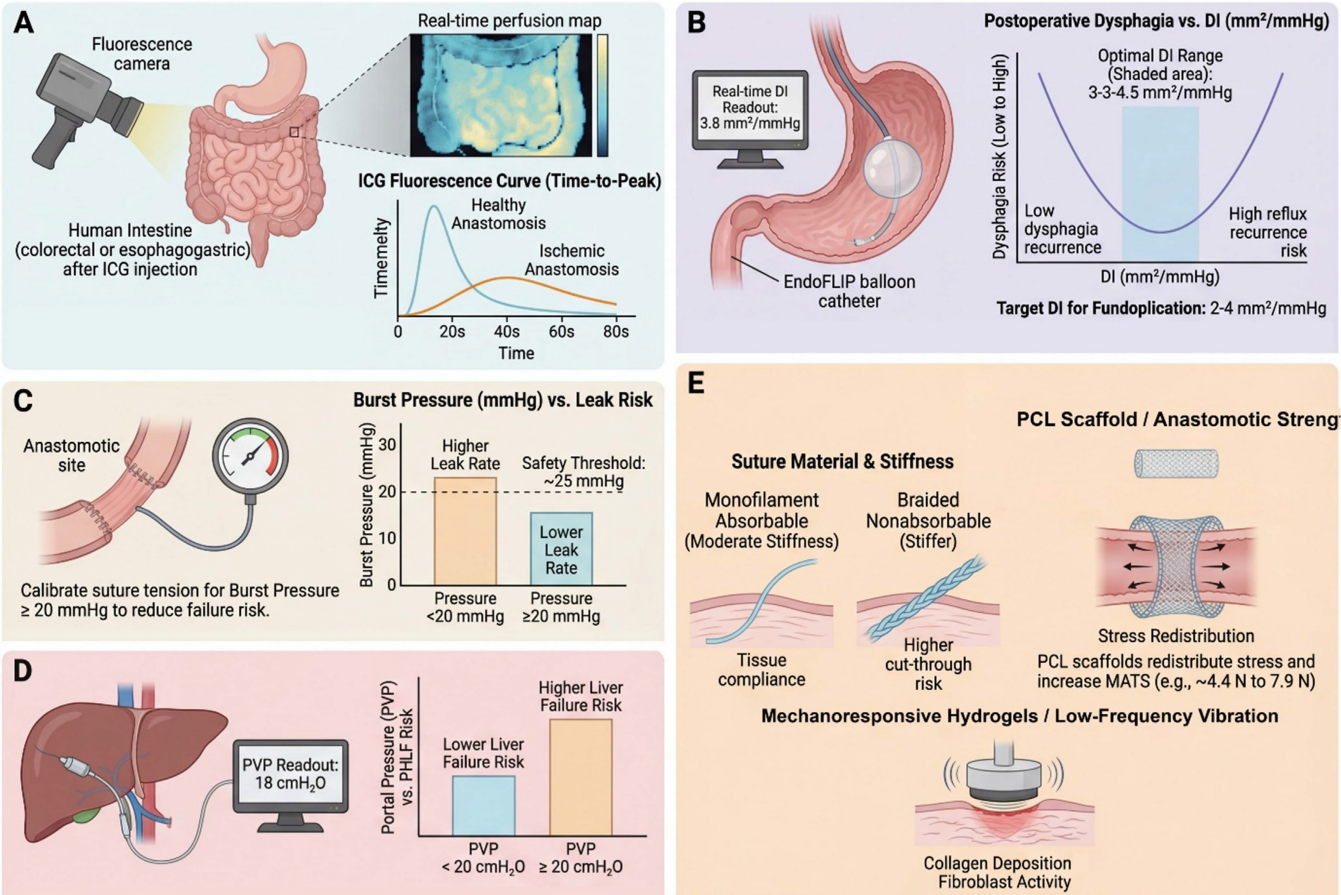
** Intraoperative mechanosensing and targeted mechanical interventions to optimize anastomotic safety and organ function. (A) Quantitative perfusion assessment of anastomotic sites.** Indocyanine green (ICG) near-infrared fluorescence is used to compare candidate transection margins in colorectal and esophagogastric surgery. Perfusion maps and time-intensity curves allow surgeons to select segments with rapid, high-amplitude uptake (for example, time-to-peak < 40 s, relative fluorescence ≥70% of reference) and avoid hypoperfused zones 59, 110. Randomized and cohort studies indicate that fluorescence-guided site selection reduces anastomotic leakage in selected or high-risk subgroups, even though overall leak rates in unselected populations may not significantly decrease 109. **(B) Functional lumen imaging and distensibility index (DI)-based titration of antireflux surgery.** Endoscopic functional lumen imaging probe (EndoFLIP) measurements during fundoplication reveal a U-shaped relationship between DI (mm^2^/mmHg) and postoperative symptoms: low DI (<1.5 at 30 mL) correlates with dysphagia, whereas very high DI (> 6.0 at 40 mL) correlates with reflux recurrence 40. Intermediate DI values (~ 3-4 at 40 mL) define an optimal window with minimal dysphagia and acceptable reflux, providing a mechanistically grounded target to guide wrap tightness and bougie size intraoperatively 53. **(C) Anastomotic tension and burst pressure thresholds.** Intraoperative pressure testing of colorectal anastomoses demonstrates that constructs failing at low pressures (< 20-25 mmHg) are more likely to leak clinically 43. Standardized air or dye leak testing at predefined pressures, combined with reinforcement of mechanically weak sites, shifts the distribution of burst pressures upward and lowers leak incidence in clinical series 108. Emerging implantable tension sensors further allow continuous quantification of loading across anastomoses as bowel distends postoperatively 38. **(D) Portal pressure-guided liver resection.** In patients undergoing major hepatectomy, intraoperatively measured portal venous pressure (PVP) serves as a physiologic readout of remnant capacity. PVP ≥ ~ 19.5 cmH_2_O after inflow control is associated with higher rates of post-hepatectomy liver failure, prompting consideration of reduced resection extent, staged approaches or adjunctive portal pressure-lowering strategies 88. **(E) Targeted intraoperative mechanical interventions.** Mechanocompatible sutures and buttressing materials are selected to match tissue stiffness and redistribute stress along anastomotic lines, reducing cutting and micro-ischemia 65, 75. Local delivery of bioactive molecules (for example, matrix metalloproteinase inhibitors or Rho-associated kinase (ROCK) inhibitors) modulates mechanotransduction and fibrosis in the early healing niche 73, 120, while low-amplitude vibration or controlled loading may beneficially prime fibroblasts and endothelial cells 129. The figure was created by Figdraw (www.figdraw.com).

Figure 4 illustrates these intraoperative strategies in tandem. The overarching goal is to ensure the anastomosis or resection bed has adequate perfusion, controlled tension, well-distributed stresses, and optimized biomaterial support—collectively reducing the risk of leaks, strictures, and other complications arising from suboptimal mechanical conditions.

## 5. Postoperative phase: mechanotherapy and personalized rehabilitation

The influence of mechanomedicine does not end when the surgery is over; the postoperative period is crucial for solidifying the success of the operation. The evidence supporting postoperative mechanotherapy also varies by maturity: early mobilization and enteral nutrition are supported by randomized controlled trials and meta-analyses; continuous IAP monitoring is supported by prospective observational studies; and mechanoresponsive drug delivery systems and implantable anastomotic biosensors remain largely experimental, with evidence from animal models and early-phase feasibility studies. In this phase, the focus shifts to promoting tissue repair, monitoring for early signs of trouble, and tailoring rehabilitation based on mechanical insights. This postoperative phase completes the theranostic cycle: continuous *in vivo* mechanical diagnostics, including intra-abdominal pressure monitoring, anastomotic stiffness tracking, and wearable biosensors, provide real-time feedback that drives personalized therapeutic adjustments. In this section, three aspects are discussed: (1) Mechanical modulation to promote healing, how controlling mechanical loads and environment after surgery (e.g., through mobilization, supports, and mechanoresponsive scaffolds) can enhance tissue repair; (2) Early warning systems for complications, novel biosensors and monitoring strategies to catch issues like leaks or compartment syndrome before they fully manifest; and (3) Long-term functional outcomes through mechanics-based rehabilitation, focusing on how the mechanical remodeling of tissues, such as anastomotic maturation, correlates with patient function, and how targeted interventions may be used to support optimal recovery.

### 5.1 Mechanical modulation to promote tissue repair

Right after surgery, an anastomosis or surgical repair is at its weakest 11. The body then initiates a healing sequence typically described as inflammation (days 1-3), proliferation (days 4-7), and remodeling (beyond the first postoperative week) 26, 30. Mechanical factors at each stage can speed up or hinder progress 37.

**Figure 5:**
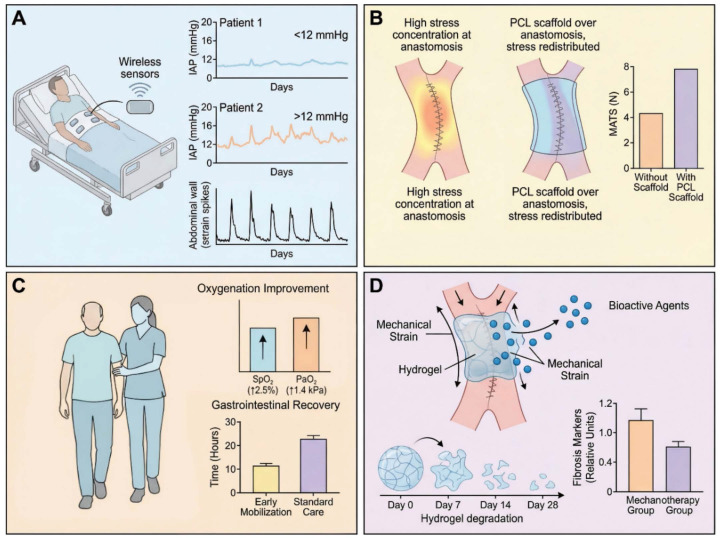
** Postoperative intelligent mechanical monitoring, force redistribution and personalized rehabilitation in digestive surgery. (A) Continuous intra-abdominal pressure (IAP) and abdominal wall tension monitoring for early detection of decompensation.** Representative IAP trajectories over the first 5 postoperative days are shown for two patients. Patient 1 maintains IAP values within the normal range (<12 mmHg), whereas Patient 2 exhibits a progressive rise into the intra-abdominal hypertension (IAH) range (≥12 mmHg) with excursions above 20 mmHg, consistent with abdominal compartment syndrome (ACS) risk 114. Parallel abdominal wall tension traces, recorded by transcutaneous stretch sensors, mirror these changes. Continuous or high-frequency monitoring via novel bladder-based or implantable pressure sensors enables threshold-based alerts and timely interventions (for example, fluid removal, decompression, loosening of external supports) before irreversible organ dysfunction occurs 143, 149. **(B) Mechanical reinforcement of anastomoses and stress redistribution.** Finite-element-style stress maps of an intestinal anastomosis highlight the mesenteric border as a biomechanical weak point with high peak tensile stress and low maximal tensile strength (MATS ≈ 4.4 ± 2.5 N under induced ischaemia). Incorporation of a poly-ε-caprolactone (PCL) scaffold redistributes stress across the circumference, lowers peak stress and increases MATS to ≈ 7.9 ± 4.2 N, thereby providing a mechanical safety margin during the vulnerable early healing period 118. **(C) Early mobilization as a systemic mechanotherapy.** In a randomized controlled trial, mobilization initiated within 2 h after major abdominal surgery improved peripheral oxygen saturation by ~ 2.5% and arterial partial pressure of oxygen by ~ 1.4 kPa compared with standard care 136. Early mobilization likely improves diaphragm excursion, lowers sustained IAP, promotes mesenteric perfusion and accelerates gastrointestinal motility, providing a low-cost, system-level mechanical intervention integrated into enhanced recovery pathways 138. **(D) Closed-loop, mechanically aware rehabilitation and drug delivery.** Postoperative care can be conceptualized as a closed-loop system in which multichannel mechanical inputs (IAP, abdominal wall tension, elastography-derived stiffness of the anastomosis) feed into a control layer that prescribes individualized adjustments in mobilization intensity, external mechanical support (for example, abdominal binders), enteral feeding regimens and local or systemic therapies 137, 145. Mechanoresponsive drug delivery systems, such as hydrogels engineered to release matrix metalloproteinase inhibitors or growth factors in response to local strain or stress, further align biochemical cues with mechanical needs 81, 151. The figure was created by Figdraw (www.figdraw.com).

One immediate consideration is stress distribution in the fresh repair 11. This issue has already been introduced in the intraoperative context, but it remains relevant postoperatively 25, 131. If one spot bears too much stress, it may tear. Research has pinpointed the mesenteric border of intestinal anastomoses as a common weak link, likely due to stress risers there 23. To address this, engineers have designed bioresorbable scaffolds or patches that can be placed over the anastomosis, particularly reinforcing the mesenteric side 118, 132. For example, a patch made of poly-*ε*-caprolactone (PCL) nanofibers has been tested in pig models: it acted like a temporary reinforcing support that distributed tension more evenly around the anastomosis. In ischemic conditions, such a scaffold increased the maximum tensile strength of the anastomosis almost two-fold at 1 week post-op from approximately 4.4 N to 7.9 N 118. The scaffold dissolves over a few months, by which time the tissue has strengthened 133, 134. This concept of force redistribution is beginning to translate clinically in other contexts too, *e.g*., internal adhesion barriers that also bear load so that healing tissues aren't directly strained. **Figure 5** will highlight such strategies.

Another key aspect is the viscoelastic nature of healing tissues 12, 62, 81. Immediately after surgery, tissues are often edematous and soft, but then gain stiffness over the following week as collagen is deposited 30, 71. It has been observed in GI anastomoses that mechanical strength rises nonlinearly over the first week, slow at first, then rapidly after day ~ 3-4 as collagen fibrils mature 11. This means that days 0-3 are a critical window where support is needed. Some surgeons use devices like vacuum-assisted closure (VAC) for difficult abdominal wounds to help approximate tissues under controlled tension, recognizing that the initial days are precarious 74. Meanwhile, tissue engineers examine the time-dependent stress relaxation of tissues. For instance, when the stomach wall is stretched, it exhibits viscoelastic relaxation, which is characterized by an initial high stress that gradually decreases under constant strain; using models like the Ogden hyperelastic model fitted to stress-relaxation tests, researchers can quantify the tissue's parameters and see how a surgical procedure such as a sleeve gastrectomy alters them 34, 36, 102. Weight loss surgery significantly reduced the elastic modulus (*E*) and yield stress of the stomach, with reports showing that elastic modulus (*E*) decreased to about 1.5 MPa after insertion, far below natural levels 34, 36, 102. This implies that the stomach wall is floppier right after such surgery, potentially delaying the return of normal motility and strength. Knowing this, one might adjust feeding regimens or add external support (like a binder) until the tissue re-stiffens with healing 11. Similarly, in colon surgery, measuring the compliance of the abdominal cavity is important 131. Low abdominal compliance, characterized by a stiff abdomen often due to obesity or prior surgeries, means any increase in intra-abdominal volume translates to a significant pressure spike, which can strain repairs 131. This underlies why obese patients with thick abdominal walls have higher risk of wound dehiscence, their abdomens don't expand easily, so internal pressure can blow out incisions 34. FE modeling studies have confirmed that adding a mesh reinforcement in the abdominal midline increases wall stiffness but also can increase overall abdominal compliance if overlapped properly, distributing forces better 135. Clinically, this suggests that prophylactic mesh in high-risk laparotomy closures may reduce hernias not just by brute force but by modulating wall mechanics 11, 135.

Early mobilization is one of the simplest and most effective mechanical interventions post-op 136. It improves oxygenation, but also influences gut motility and intra-abdominal pressure patterns 136, 137. Early mobilization prevents the persistently elevated intra-abdominal pressure as observed in bedridden patients, a phenomenon attributable to factors such as diaphragm splinting and ileus 114. Enhanced Recovery After Surgery (ERAS) protocols universally include early mobilization for these reasons, which is supported by findings that even sitting in a chair for a few hours on the day of surgery leads to improved pulmonary function and potentially faster return of bowel function 136. Another study in high-risk abdominal surgery patients found that more than 80% of patients were able to complete four mobilization sessions per day by postoperative day 3, which correlated with shorter length of stay 138. Mechanistically, early mobilization likely stimulates mesenteric circulation and the release of growth factors or hormones that facilitate healing, with supporting data showing that exercise can increase VEGF levels and improve collagen deposition quality 54, 58. It also conditions the abdominal wall, preventing stiffening from inactivity 131.

Nutritional support has a mechanical angle too 139. Early enteral feeding, in contrast to maintaining a nil-by-mouth (NPO) status or total parenteral nutrition, promotes gastrointestinal motility; the resulting peristaltic activity imposes cyclical mechanical stresses on the anastomosis, delivering a stimulus that is both gentle and physiologically normative 79, 140. This may actually strengthen the repair by encouraging normal tissue remodeling as opposed to stasis 25. Additionally, luminal nutrition ensures that the mucosa, which is one of the fastest-healing layers, recovers quickly to seal the inside 139. Studies in critical care have shown benefits of enteral over parenteral nutrition in outcomes, partly attributed to maintenance of gut integrity 140, 141. Conversely, excessively aggressive feeding may increase luminal pressure or overdistension, and therefore a balanced approach is required 137. Fluid dynamics of chyme are considered: a high-volume, low-viscosity feed might cause more turbulent flow and shear stress in the gut; therefore, starting with small, frequent, nutrient-dense feeds (which have higher viscosity) could impose less shear stress on the anastomosis initially 42. Using Caco-2 cells as an intestinal epithelial model, experiments established a beneficial shear stress range of 0.2-0.5 Pa for promoting mucus secretion and barrier function, with deviations to either extreme being detrimental 49, 50. A moderate flow (as occurs with trickle feeds) may actually encourage the epithelium to strengthen, whereas no flow (ileus) or extremely high flow (diarrhea) could be harmful to healing 49. So, practically, starting enteral feeds early at a slow rate aligns with this principle, providing that gentle mechanical stimulation along with nutrition 137, 140.

All these strategies, reinforcement devices, mobilization, careful feeding, are forms of mechanotherapy: treating the patient by modulating mechanical conditions 37. They can be thought of as “prescriptions” just like medications. In some places, physical therapists now work with surgical patients on the first day after surgery to mobilize them, essentially prescribing a specific mechanical activity as therapy 136, 138. Abdominal binders are another simple tool: by supporting the abdominal wall, they can reduce the force on incisions with coughing or movement 135. However, binders also increase external constraint, so one must not overtighten which would raise baseline IAP 137, 142. It is a fine example of how subtle mechanical nuances matter, a binder snug enough to support but not so tight as to elevate pressure.

### 5.2 Early warning and proactive mechanical intervention for complications

Despite these efforts, complications can still occur; early detection and prompt intervention are therefore crucial 2, 4. Mechanomedicine contributes here by providing continuous *in vivo* diagnostic monitoring systems and criteria for early intervention based on mechanical signals 37. The postoperative biosensor ecosystem, encompassing implantable pressure capsules, wearable abdominal strain sensors, and wireless motility trackers, constitutes a distributed theranostic network in which each diagnostic signal is coupled to a predefined therapeutic response protocol.

One of the clearest cases is intra-abdominal hypertension (IAH) and abdominal compartment syndrome (ACS) after major surgery 114, 137. If fluid accumulates or organs swell significantly, IAP can rise to dangerous levels, impairing organ perfusion and threatening anastomoses 131. Traditionally, IAP is measured intermittently via the bladder manometry technique 142. Continuous or high-frequency IAP monitoring devices are emerging 143. For example, a wireless bladder pressure biosensor has been developed that sits in the Foley catheter and transmits real-time bladder pressure (which reflects IAP) 143. There are also implantable pressure capsules, conceptually similar to the “SmartPill” but designed for the peritoneal cavity, which can provide constant telemetric pressure readings 144. These devices represent miniaturized *in vivo* diagnostic biosensors that embody the theranostic principle and could potentially support alert-based intervention protocols once validated.

For anastomotic leak detection, mechanical and biochemical *in vivo* diagnostic monitoring holds promise but remains largely experimental 95, 96. One approach is measuring transanastomotic impedance or pressure changes 38. A healing anastomosis, if intact, often exhibits a certain pattern: slight increase in luminal pressure when peristalsis hits it, and no free air leak. If a dehiscence begins, abnormal pressure fluctuations or a loss of expected resistance may be observed 25, 79. In fact, one device currently in trials is a nanoparticle-based biosensor film that changes electrical resistance in response to a local pH change, a change that occurs upon exposure to leaking bowel contents 145-147. This nanobiosensor exemplifies the theranostic potential of implantable diagnostic devices: it provides continuous *in vivo* monitoring of anastomotic integrity and, in future iterations, could be coupled to a drug-releasing layer that responds to the same pH signal by locally delivering antimicrobials or sealants, thereby integrating diagnosis and therapy in a single implantable platform. Another simpler measure is abdominal wall tension: if an anastomotic leak begins, often ileus and fluid collections develop, subtly increasing IAP and causing abdominal wall strain 131. A noninvasive device fitted with abdominal stretch sensors can infer elevations in intra-abdominal pressure by monitoring changes in abdominal wall strain 142. A pilot study reported a strong correlation (*r* ~ 0.85) between abdominal surface strain and bladder pressure in postoperative patients. In principle, such a device could be used on the ward to flag rapid increases in abdominal wall strain or girth that may warrant further evaluation for ileus, fluid accumulation, hemorrhage, or leak 142. These wearable and implantable sensing approaches are still at an early experimental stage, but the direction is clear: mechanical vital signs, such as intra-abdominal pressure, abdominal wall tension, and organ movement, will become part of the standard monitoring parameters for postoperative patients, alongside heart rate and temperature 148-150.

Another technology is the “SmartPill” intra-abdominal sensor capsule mentioned above 144. A version of this can measure pressure from 0 to 30 mmHg with 0.1 mmHg resolution and respond within < 1 second 144. If placed near an anastomosis, such a device could theoretically detect pressure spikes or vibrations indicative of a leak, which are caused by the distinct pressure pattern generated as air bubbles pass through a defect 25. Integration of such data into AI-assisted monitoring systems could improve early detection of anastomotic leaks. At present, clinical recognition still relies mainly on delayed signs such as fever, tachycardia, peritonitis, or intermittent imaging assessment 84, 95. These implantable biosensor platforms, combining point-of-care *in vivo* diagnostics with AI-driven interpretation, represent a new generation of theranostic devices for postoperative care.

Another area where mechanical early warning is useful is ileus vs. obstruction differentiation in the days after surgery 39. Everyone expects some ileus post-op, but differentiating a prolonged ileus from an early adhesive obstruction is hard 25. The use of mechanical monitoring, for instance via a wireless motility capsule to measure intraluminal pressures and motility, could enable the differentiation of coordinated high-pressure patterns characteristic of obstructive pathologies from the patterns indicative of an atonic ileus 79, 144. Hypothetically, future systems may define quantitative motility thresholds for distinguishing prolonged ileus from early mechanical obstruction.

Lastly, consider intraoperatively applied anti-adhesion materials that provide preventive mechanical benefits during the postoperative period, such as PuraStat, a hemostatic peptide gel 121. These not only help hemostasis but can also serve a mechanical function: they create a protective layer that reduces friction and shear between organs as they slide postoperatively 121, 151. PuraStat, for instance, has a measured elastic modulus tuned to mimic peritoneal tissue, allowing it to move with adjacent tissues rather than generating additional shear at the interface 121. Related studies have shown that it reduced adhesion formation in a rat model, presumably because it absorbed mechanical stresses that would otherwise stimulate adhesion-forming cells 152, 153. While this is more preventive than an “early warning”, it shows another proactive mechanical strategy: physically intervening to alter how tissues interface in the healing period 37, 154.

In summary, the postoperative period benefits from a proactive, surveillance mindset, using mechanical cues as early indicators and intervening before a problem becomes full-blown 37. This strategy mirrors the evolution in cardiology from treating infarctions to preempting them by monitoring ischemia. In surgery, it entails detecting abnormal mechanical signatures, namely pathological pressure or strain, that foreshadow a leak, rather than waiting for clinical decline 95. Implementing these requires interdisciplinary collaboration, surgeons, engineers, critical care physicians, to validate that these new monitors and devices truly correlate with meaningful endpoints 83, 84. However, recent trends, including evolving regulatory pathways for such devices are evolving and a growing volume of clinical studies, highlight the potential of this technology 155, 156. With the emergence of accurate continuous IAP monitoring systems, there is potential for this mechanical parameter to become a key diagnostic tool in the perioperative management of high-risk surgical patients, addressing the current limitations in diagnosing abdominal compartment syndrome.

### 5.3 Long-term functional outcomes and mechanics-based rehabilitation

Even after the immediate recovery phase, the mechanical microenvironment continues to influence long-term outcomes 37, 71. Patients often care most about functional recovery, e.g., regaining normal bowel function, avoiding strictures or hernias, and returning to daily activities 56. Long-term outcomes are closely related to how tissues remodel mechanically in the weeks and months after surgery 30, 72. Long-term mechanical monitoring, using outpatient elastography and wearable biosensors, thus extends the theranostic cycle beyond the hospital, enabling personalized, data-driven rehabilitation that adjusts therapeutic interventions based on ongoing diagnostic measurements of tissue remodeling.

Consider an intestinal anastomosis months later, it should ideally exhibit compliance similar to that of the adjacent bowel so that peristalsis propels contents smoothly 25. If it becomes a rigid scar ring (high stiffness), the patient might develop a stricture and obstructive symptoms 56, 69. Thus, monitoring the mechanical properties of healing tissues in the longer term can identify who's at risk of such issues 20. Techniques like ultrasound strain elastography or SWE can be used even after a patient has left the hospital, to measure anastomosis stiffness during follow-ups 20, 91. In the context of Crohn's disease, SWE measures stiffness in strictures, with higher values signifying a fibrotic phenotype suggestive of a need for surgery, thereby distinguishing it from an inflammatory stricture amenable to medical treatment 20. Similarly, a healing colorectal anastomosis that shows increasing stiffness over two months might indicate excessive fibrosis, prompting a potential intervention such as endoscopic dilation or the use of anti-fibrotic medications to prevent full narrowing 56. Emerging evidence suggests that fibrotic remodeling is driven by reciprocal interactions between mechanical cues and profibrotic signaling pathways, including TGF-β/Smads, Hippo-YAP/TAZ, and mechanosensitive channels such as TRPV4, highlighting these pathways as potential targets for future personalized anti-fibrotic interventions in patients with mechanically high-risk healing phenotypes 58, 71, 80.

Another concept introduced is the Mechanical Compatibility Index (MCI) of an anastomosis. This index could combine measures of stiffness uniformity and strength 11. A perfectly integrated anastomosis would have a MCI approaching 1, denoting that it is mechanically indistinguishable from the native surrounding tissue 12. If the anastomosis is too stiff or too weak relative to adjacent tissue, the MCI deviates from the ideal, a deviation which one study proposed to quantify with a formula based on the ratios of its elastic modulus and tensile strength to those of normal tissue 148. They found that an MCI within a specific range predicted the return of normal bowel function, whereas a low MCI predicted dysfunction due to the anastomosis being either excessively compliant or overly stiff 64, 148, 157. This kind of quantitative metric is reminiscent of how cardiologists quantify valve function or graft patency; surgeons might do the same for repairs.

Long-term, patients worry about things like incisional hernias after abdominal surgery 135. Mechanically, an incisional hernia is a failure of the abdominal wall to withstand internal pressure 131, 158. From a mechanistic perspective, this may reflect reduced collagen strength or uneven force distribution, or maybe the distribution of forces across their closure was uneven 135. Some surgeons proactively reinforce high-risk incisions with mesh to offload forces, while follow-up imaging or even dynamic MRI can assess how the abdominal wall moves and strains under Valsalva, enabling differentiation between patients with abnormal bulge and high strain at the scar who may benefit from earlier hernia repair versus those whose wall remains stable 131, 135.

Adhesions are another long-term outcome 121. Mechanically, adhesions form when scar bridges between surfaces that move relative to each other 74. Reduction of friction, for example through adhesion barriers, or short-term reduction of relative motion between surfaces may lessen adhesion formation. Conversely, too much motion early might cause micro-bleeds and inflammation leading to adhesions 26. An appropriate mechanical balance is required, in which complete immobilization is avoided while unnecessary abrasion is minimized through microsurgical gentle technique and copious irrigation to prevent dry surfaces from scraping 121.

It is worth noting the interplay of mechanics and ongoing biology: radiation therapy after surgery can stiffen tissues through radiation fibrosis 71, 80. Patients who get pelvic radiation post resection might have stricter, less compliant anastomoses and higher risk of bowel dysfunction 157. Recognizing this risk, clinicians might employ more aggressive dilation or medical therapy to mitigate radiation fibrosis, such as pentoxifylline and vitamin E, which some oncologists use for radiation proctitis to soften tissues 71. Again, mechanomedicine provides the rationale: radiation injures microvasculature and causes collagen crosslinking, a mechanical issue 37.

Finally, patient-specific factors like age and activity level matter 74. Older patients heal more slowly and often develop stiffer scars with less elastic collagen 26. Encouraging appropriate exercise, such as light core exercises a few weeks after surgery, could stimulate better remodeling of the abdominal wall scar, analogous to Wolff's law for fascia 136. Younger patients might exhibit excessive healing responses, including keloids and exuberant scars; modulating the mechanical microenvironment by avoiding premature scar overloading could potentially prevent such outcomes 74.

In essence, just as physical therapy is essential after orthopedic surgery to ensure optimal function, mechanical rehabilitation after abdominal surgery can be viewed as a continual process of monitoring surgical repair integration and adjusting patient activities or applying therapies to guide that integration. Advanced imaging and sensors might be employed to ensure that by three months postoperatively, the stiffness of the repair approximates native tissue, or if discrepancies exist, to implement targeted interventions such as endoscopic stricturotomy for excessive stiffness or supportive abdominal binders for insufficient tension.

**Table 2** summarizes the modulation strategies across the preoperative, intraoperative, and postoperative stages.

By continuously linking mechanical parameters to clinical decisions, from start to finish, the patient's journey becomes one of mechanically-informed care 37. The long-term vision is that surgeons will not only operate but also guide tissue healing toward optimal integration, using data and devices to ensure that at 6 months postoperatively, the patient's tissues are as functional as possible 136. This approach should reduce late complications like strictures, hernias, or adhesions that often necessitate reoperations or cause chronic issues 56, 135.

## 6. Future directions and challenges in mechanomedicine

As mechanomedicine in digestive surgery is an emerging field, significant innovations and challenges lie ahead. Several key future directions are outlined below, focusing on the integration of emerging technologies, the need for standardizing mechanical measures in clinical practice, and addressing foundational scientific questions that remain unresolved.

A central theme across all future directions is the progressive realization of a fully integrated theranostic system, in which *in vitro* diagnostic platforms, *in vivo* biosensors, and mechanoresponsive therapeutic devices are seamlessly connected through AI-driven decision layers.

### 6.1 Integrating emerging technologies for precision surgery

The coming years will likely see an even deeper convergence of mechanomedicine with advanced technologies such as AI, robotics, and biofabrication 37, 83. On the intraoperative front, the rise of surgical robotics and intelligent assistance systems provides an ideal platform to incorporate mechanical diagnostic-therapeutic feedback loops. For instance, next-generation robotic surgery systems are being developed with built-in force biosensors and AI-driven controllers that can maintain optimal tension on tissues automatically. Related reports demonstrated a robot that could adjust its grasping force on bowel tissue in real time to avoid excessive stress, using reinforcement learning to learn the delicate balance 38, 103. This hints at a future where the surgeon sets a target tension range, and the robot ensures that is not exceeded as it sutures, effectively embedding theranostic mechanomedicine algorithms into the robotic workflow. Such robotic theranostic systems, in which force biosensors continuously monitor the mechanical state of tissue and actuators simultaneously deliver the appropriate therapeutic response, could represent an advanced form of diagnostic-therapeutic integration.

AI-driven planning and guidance will also advance substantially. Mechanics-aware AI systems may eventually provide recommendations that extend beyond anatomical image analysis. For example, such platforms may help predict where high-stress regions are likely to arise under a given surgical approach. This capability could be integrated into augmented reality displays for surgeons, perhaps presenting a color overlay on organs indicating stiffness variations or risk zones, such as a red band showing areas where tension would be excessive if transected. Preliminary versions of this concept have been reported: one system analyzed liver elastography data and projected a stiffness map onto the surgeon's augmented reality glasses during liver resection to identify and avoid highly stiff, cirrhotic areas that might not heal well 21, 159.

Miniaturized, wireless biosensors—currently at the proof-of-concept stage—represent a major component of future theranostic integration. Building on implantable and wearable technologies discussed previously, future iterations will be increasingly sophisticated, possibly powered by the body through thermoelectric or piezoelectric conversion of movement-derived energy and networked via Internet of Things infrastructure. A patient recovering at home could have a tiny implanted intra-abdominal pressure biosensor and a wearable gut motility tracker transmitting data to a smartphone application that uses artificial intelligence to interpret signals and provide simplified guidance indicating either stable recovery or the need for clinical evaluation. Recent advances in implantable flexible and soft mechanical sensors suggest that wireless, mechanically conformable, and potentially bioresorbable platforms for *in vivo* monitoring are becoming increasingly feasible, laying the groundwork for transient postoperative biosensors that could integrate sensing, communication, and eventual resorption 160, 161. Imagine a theranostic implant placed at an anastomosis that monitors mechanical integrity for 2 weeks, triggers local drug release if abnormal signals are detected, and then dissolves harmlessly once healing is confirmed. This vision of a bioresorbable, self-reporting, self-treating theranostic implant represents the convergence of nanobiosensor technology, bioelectronics, and mechanoresponsive drug delivery that defines the frontier of the field.

3D and 4D printing for patient-specific implants and supports is poised to expand significantly. Currently, three-dimensional-printed ostomy supports, custom hernia meshes precisely shaped to a patient's anatomical defect, and bioresorbable three-dimensional printed stents for gastrointestinal anastomoses are already being used to maintain luminal patency before they resorb 127, 128. The next step is the development of 4D-printed devices, which are designed to change their properties over time. For example, a 4D-printed anastomotic ring that is initially stiff to provide support but gradually softens after 2 weeks to avoid obstruction, and then fully resorbs by 6 weeks when healing is solid. Such smart implants could adapt to the healing stage, something not possible with static materials. In oncology, patient-tailored 3D-printed scaffolds might be used after tumor resection to both replace tissue and deliver localized therapy, for example, a printed patch for the abdominal wall that releases chemotherapy to prevent peritoneal metastases, while mechanically reinforcing the wall.

All these integrations are directed towards a single fundamental goal: precision and personalization. Mechanomedicine provides the data framework and therapeutic targets, such as maintaining tension below a specified threshold and stiffness above a minimum value, while technologies including artificial intelligence and robotics provide the means to achieve these targets with superhuman consistency. As these tools mature, surgeries will likely become safer, with fewer human errors, greater adaptability, and more predictable outcomes. Human oversight and clinical judgment remain essential; however, the substantial work of simultaneously monitoring numerous parameters and making micro-adjustments can be delegated to machines that excel at such tasks.

In summary, the operating room of the future might incorporate robotic arms that sense and respond to tissue properties, augmented reality for visualizing occult mechanical properties, artificial intelligence-driven copilots predicting complication risks in real time, and patient-specific implants fabricated on-demand to ensure each patient's tissue receives precisely calibrated mechanical support. Early versions of these technologies already exist, some in research phases and others in early clinical trials 155, 156. The integration of mechanomedicine with such technologies will drive a new era of “digital mechanosurgery”, where data-driven and automated systems work alongside surgeons for optimal outcomes.

### 6.2 Standardizing mechanical metrics and guidelines

One major challenge for bringing mechanomedicine fully into routine practice is the lack of standardized reference values and guidelines. Unlike blood pressure or blood glucose, where well-defined normal ranges and treatment thresholds are available, mechanical parameters in surgery, such as normal liver stiffness for a 60-year-old patient or safe anastomotic tension ranges, are not yet universally established 12, 159. This hampers comparability and broad adoption.

For example, different elastography machines might report slightly different absolute stiffness values for the same liver. A stiffness of 10 kPa on one device might correspond to 8 kPa on another due to calibration differences. Thus, a key step is creating cross-platform calibration standards. Phantom materials mimicking tissue stiffness need to be standardized so that MRE at Hospital A and SWE at Hospital B can both calibrate to the same reference. Organizations could then publish reference stiffness ranges for various organs stratified by factors such as age and body mass index, since even normal stiffness might vary with these parameters. Indeed, a global consortium on elastography has been called for, analogous to an internationally harmonized stiffness reference framework.

Similarly, for intraoperative measures, one center might aim for a DI of 3-4 in fundoplication while another uses a different device and thus different numerical targets. If standardized protocols are universally adopted, such as a standard EndoFLIP protocol involving 40 mL fill volume and measurement of DI at fundoplication closure, then guidelines can recommend ensuring fundoplication DI between 3-4 mm^2^/mmHg may eventually support formal guideline recommendations, allowing all surgeons to train to that standard. This requires professional societies to endorse these metrics and provide appropriate training.

Another area is developing clinical guidelines that incorporate mechanical metrics. For instance, an “Enhanced Recovery Mechanomedicine Protocol” could be developed to specify the following: measure IAP every 8 hours for first 3 days, if > 12 mmHg initiate such-and-such 137, 143; measure liver stiffness preoperative in major hepatectomy, if > 15 kPa do these additional steps 21, 67; measure colorectal anastomotic perfusion with ICG, if time-to-peak exceeds 30 s, consideration may be given to creation of a stoma 59, 162. In the future, specialty guidelines may incorporate elastography into hepatobiliary surgical assessment and include ICG-based perfusion evaluation in colorectal surgical guidance, provided that stronger multicenter evidence becomes available 90, 109.

To build that evidence, large databases need to collect mechanical data alongside outcomes. A proposal has been made for a multilayer mechanical parameter registry collecting data at the organ level, such as stiffness of liver and intestine in various conditions; at the pathology level, tracking how stiffness changes from normal to mild fibrosis to severe fibrosis; and at the healing phase level, tracking anastomotic tension over time and correlating with leak occurrence 71, 104. Such datasets could support the development of quantitative risk models relating anastomotic tension to leak probability.

Another need is defining a “mechanical dose” in surgery, analogous to radiation dose in radiotherapy 12, 22. At present, anastomotic tension is often described qualitatively rather than quantified using standardized mechanical dose metrics 23, 43. Some have proposed quantifying the energy density at the anastomosis, potentially as the integral of stress-strain over time, as a dose metric 22, 45, 64. Definition of a threshold mechanical dose would allow clinical anastomoses to be maintained below that level. This remains challenging because tissues exhibit nonlinear, patient-specific behavior, but this concept might see development, especially as sensors provide more real-time data to calculate such values.

Finally, training and culture is a part of standardization. Surgeons will need to become as comfortable with reading an elastogram or interpreting a sensor readout as they are with reading an electrocardiogram 159, 163. That means integrating this education into surgical training curricula. The more data and guidelines emphasize these, the more surgeons will adopt them. It's analogous to the adoption of laparoscopy, at first some resisted, but once guidelines and training incorporated it, it became second nature.

In summary, to fully leverage mechanomedicine, a common language and standardized benchmarks are needed: reference ranges for mechanical measures, agreed protocols for measurement, and guidelines for acting on those measures. Given the importance of this issue, it is anticipated that, over the next 5 years, surgical societies and regulatory bodies such as the FDA will increasingly mandate reporting of certain mechanical metrics in device trials or surgical audits, which will naturally promote standardization. Early movements include the FDA's recent guidance on brain compliance monitors and the European consensus on SWE for liver disease, which defined cutoff values for fibrosis stages 164, 165. As these accumulate, mechanomedicine will move from interesting research to everyday practice.

### 6.3 Addressing foundational scientific questions

While applying current knowledge is crucial, there remain fundamental scientific questions in mechanomedicine that require further research. Addressing these questions will reduce uncertainty and facilitate further progress in the field 12, 37.

Inverse problems in biomechanics represent a significant challenge in determining how accurately true tissue properties can be inferred from measurements such as elastography waves 44, 166. For example, MRE inversion algorithms attempt to compute the shear modulus from observed wave fields 167, 168. But under conditions of limited field of view or noise, there are identifiability limits, meaning that there are thresholds beyond which a given measurement becomes unreliable in distinguishing a change in stiffness vs. an artifact 169, 170. Research here overlaps with applied math and physics: establishing error bounds and robust inversion methods 166. Such uncertainty limits reproducibility across platforms and remains a major barrier to clinical standardization. Machine learning-based inversion also introduces challenges related to interpretability, generalization, and training-data bias. Recent work has improved inversion algorithms by incorporating machine learning, essentially training on simulated wave data to better decode overlapping waves in small organs such as the pancreas. However, more work is needed, and multi-modality approaches combining elastography with imaging constraints will likely prove beneficial.

Another fundamental area is multi-scale mapping: linking micro-scale properties, such as ECM fiber organization, to macro-scale properties, such as organ compliance. It is well established that collagen crosslinking increases stiffness qualitatively, but predictive models are desirable: for instance, given a biopsy with specific collagen percentage and crosslink density, one could predict liver stiffness and portal pressure for that patient. Some progress has been made using micro-computed tomography of resected tissues to model ECM networks; recent studies have shown a direct correlation between collagen fiber alignment in intestinal strictures and the degree of nonlinearity in their stress-strain response 51, 56. Eventually, an “ECM fingerprint” may become available to indicate which helps in creating personalized models without having to individually measure everything *in vivo*.

A highly intriguing concept involves defining an “anastomotic mechanical dose”, analogous to radiation dose definition in radiation oncology. Surgeons might quantify how much mechanical stress an anastomosis endures over time as its dose, which correlates with healing or complication rates. For instance, one could integrate the tension over each day for the first postoperative week using some function of magnitude and duration. One possibility is that leakage risk increases once a threshold dose is exceeded. Early porcine studies have attempted to relate time under tension to leak occurrence, although the concept remains incompletely defined. This likely requires a time-dependent model of tissue strength gain versus applied stress, essentially a dynamic fracture mechanics model for living tissue. Answering this question requires both computational modeling and animal studies in which the mechanical microenvironment is tightly controlled to observe when failure occurs. Solving this would represent a paradigm shift: surgeons could then aim to keep mechanical dose below a certain level through rest, dietary management, supportive measures, and other interventions until tissue strength adequately increases, representing a highly quantitative approach to the principle of “don't stress the anastomosis too much too soon.”

Another challenge involves maintaining stability and robustness in closed-loop control during surgery. If such a system is implemented, care must be taken to avoid over-correction or oscillation, which are classic control theory issues. Additionally, considering the human element, surgeons must trust and effectively interact with these systems. Research analogous to autopilot systems in aviation is needed: developing fail-safes, alarm hierarchies determining when the machine versus the human takes control, and proving that these closed-loop systems actually improve outcomes. Early closed-loop trials exist in anesthesia, such as closed-loop propofol infusion controlling bispectral index brain monitoring. Closed-loop fluid management may emerge to maintain optimal abdominal compliance or closed-loop insufflator systems adjusting pressure to maintain target indocyanine green perfusion metrics. However, rigorous trials are needed to demonstrate safety and benefit, and those are on the horizon.

Finally, broad adoption will also depend on demonstrating cost-effectiveness and training surgeons in these new paradigms. This represents more health-services research but constitutes a necessary component of answering the question: is mechanomedicine worth implementing? If it reduces complication rates, which are highly costly, the balance may ultimately prove favorable, although this will require confirmation in multicenter studies.

### 6.4 Data Ethics, Equity, and Responsible Implementation

The integration of AI and the Internet of Things into mechanomedicine raises important ethical and equity considerations that must be addressed alongside technological development. First, data privacy and governance: continuous biosensors, wearable devices, and AI-driven monitoring systems generate large volumes of sensitive patient data. Robust, transparent data governance frameworks—including informed consent protocols, data anonymization standards, and secure storage architectures—are essential to protect patient privacy and maintain public trust. Second, algorithmic bias and generalizability: AI models trained predominantly on data from high-resource settings or specific demographic groups may perform poorly when applied to diverse populations. Future research must rigorously evaluate the generalizability and fairness of algorithmic models across different ethnicities, ages, body habitus, and healthcare settings, and actively work to mitigate bias in training datasets. Third, healthcare equity: advanced mechanomedicine technologies, including high-field MRE systems, robotic surgical platforms, and implantable biosensors, are currently concentrated in well-resourced academic centers. Without deliberate efforts to reduce cost, simplify deployment, and expand access, these innovations risk exacerbating existing healthcare inequalities between high- and low-income settings. Responsible implementation of mechanomedicine therefore requires not only technical validation and clinical translation, but also a commitment to equitable access, transparent algorithmic accountability, and inclusive research design.

**Figure 6:**
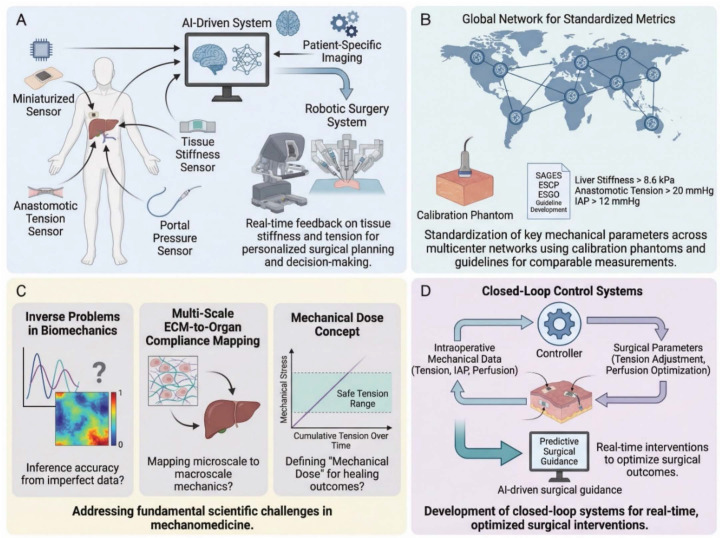
** Future scientific challenges and translational roadmap for mechanomedicine in digestive surgery.** This figure outlines a forward-looking framework linking sensing technologies, biomechanical modeling, standardization efforts and control strategies to enable precision mechanomedicine in digestive surgery. **(A) Integrating sensing, modeling and artificial intelligence for precision mechanosurgery.** The technological trajectory of mechanomedicine involves three tightly coupled layers: (1) miniaturized, biocompatible sensing and imaging modalities, including elastography, functional lumen imaging, implantable tension and pressure sensors, and wearable abdominal patches, which provide high-frequency, quantitative measurements of tissue stiffness, tension and pressure *in vivo* 144, 146; (2) data-fusion and modeling engines that integrate physics-based finite-element (FE) simulations with machine learning algorithms and multimodal data streams to infer latent mechanical states, predict patient-specific risk and support surgical decision-making 85, 105; (3) clinical interfaces, such as robotic surgical consoles, augmented reality overlays and bedside dashboards, that deliver mechanorelevant information to surgeons and clinicians in real time and enable adaptive, personalized intervention strategies 55. **(B) Standardizing mechanical metrics and clinical practice across centers.** For mechanomedicine to transition from research to routine care, mechanical parameters must be harmonized across institutions, devices and vendors. This requires: the establishment of multicentre registries of organ and lesion stiffness, stratified by age, sex, body-mass index and disease stage 168; phantom-based calibration frameworks for elastography, EndoFLIP and related technologies to ensure cross-platform and cross-vendor comparability 165; and the incorporation of validated mechanical thresholds—such as liver stiffness cut-offs for hepatectomy, distensibility index windows for fundoplication, or intra-abdominal pressure (IAP) triggers for decompression—into international clinical guidelines and consensus statements 159. **(C) Open scientific questions in mechanomedicine.** Key unresolved challenges include: defining the identifiability limits of shear modulus (μ) and elastic modulus (E) from noisy and incomplete wave-field data in organs with heterogeneous composition and complex geometry 166; establishing a quantitative, multiscale mapping from extracellular-matrix architecture and cellular organization to organ-level compliance and distensibility indices, enabling mechanistic interpretation of clinical cut-offs 63; and formulating a clinically meaningful concept of an anastomotic “mechanical dose”, for example as time-integrated stress, strain or strain-energy density, and linking it to outcomes such as leak, fibrosis and stricture formation 69. **(D) Closed-loop control systems for real-time surgical optimization.** Building on standardized metrics and mechanistic models, closed-loop control architectures can be developed in which intraoperative mechanical measurements (such as tissue tension, distensibility index, IAP and perfusion metrics derived from indocyanine green imaging) are continuously fed into a controller that adjusts surgical parameters—including tension, perfusion optimization and decompression strategies—in real time. AI-driven predictive guidance layers can further enhance robustness and personalization, enabling stable, adaptive control across heterogeneous patient populations and clinical settings. Together, addressing these scientific, technological and translational challenges—alongside parallel regulatory, ethical and educational initiatives—will be essential to evolve mechanomedicine from promising prototypes into a mature, globally adopted pillar of digestive surgery 155, 164. The figure was created by Figdraw (www.figdraw.com).

In summary, future progress in mechanomedicine will rely on sustained interdisciplinary collaboration among surgeons, engineers, biologists, data scientists, and other relevant specialists. Addressing current challenges will require both technical innovation and effective integration across disciplines. Over the coming decade, several emerging concepts may move toward routine clinical application, supported by growing evidence and increasing clinical interest. The long-term goal is a model of digestive surgery in which clinical decisions are guided by a deeper understanding of tissue mechanics, perioperative care is personalized through mechanical profiling, and adverse outcomes are reduced through more precise intervention. Figure 6 summarizes the major scientific challenges and outlines a translational roadmap for mechanomedicine in digestive surgery, including sensing-modeling integration, standardization, and closed-loop control strategies.

## 7. Conclusions

Mechanomedicine is reshaping digestive surgery by bringing the mechanical microenvironment into the center of perioperative care. Mechanical parameters such as tissue stiffness, anastomotic tension, perfusion-related forces, and intra-abdominal pressure are no longer merely descriptive features but increasingly actionable variables for risk stratification, surgical guidance, and postoperative monitoring.

This review highlights a unifying concept: across the perioperative continuum, mechanical information can link diagnosis with intervention and thereby support a more quantitative and individualized surgical strategy. Preoperative profiling, intraoperative mechanosensing, and postoperative mechanotherapy together outline a framework in which surgical outcomes may be improved not only by anatomical precision, but also by better control of tissue mechanics during healing and remodeling.

At present, mechanomedicine remains an evolving field. Some tools have already shown clinical utility, whereas others still require further validation, standardization, and integration into routine workflows. Nevertheless, the overall direction is clear: incorporating biomechanics into digestive surgery offers a promising path toward more predictive, precise, and personalized care.

## Figures and Tables

**Table 1:** Representative mechanical parameters, measurement modalities, and clinical scenarios in digestive surgery

Mechanical dimension	Representative quantity	Typical values or thresholds	Assessment modality	Corresponding clinical scenario	Refs
Liver stiffness	Elastography-derived stiffness (kPa)	LS < 8.6 kPa: lower-risk hepatectomy candidacy; LS > 12-15 kPa: advanced fibrosis/high-risk liver reserve (C/T)	MRE; 2D-SWE	Preoperative candidacy assessment and planning for liver resection	12, 67, 68, 86
Pancreatic stiffness	SWV / elastography-derived stiffness	Low stiffness (e.g., SWV < 1.3 m/s) associated with higher POPF risk (T)	SWE; ultrasound elastography	Preoperative risk assessment before pancreatoduodenectomy	19, 46, 47
Lumen distensibility	DI = cross-sectional area / intraluminal pressure (mm^2^/mmHg)	Low DI indicates an overly tight wrap; target ranges are procedure-dependent, often about 2-4 mm^2^/mmHg or > 3.5 mm^2^/mmHg at 40 mL (C/T)	EndoFLIP	Intraoperative assessment and adjustment of fundoplication	40, 53, 107
Perfusion / microcirculation	Quantitative fluorescence parameters	Delayed fluorescence or weak intensity suggests impaired perfusion (T)	Quantitative ICG fluorescence imaging	Intraoperative bowel/conduit perfusion assessment and anastomotic site selection	59, 109, 110, 158
Portal venous pressure	PVP	PVP ≥ 20 mmHg after clamping/resection indicates high risk in selected studies (T)	Direct intraoperative portal pressure measurement	Intraoperative hemodynamic assessment during major hepatectomy	88
Intra-abdominal pressure	IAP	IAH: IAP ≥ 12 mmHg; ACS: IAP ≥ 20 mmHg (C)	Bladder pressure monitoring; continuous IAP monitoring	Perioperative and postoperative surveillance after major abdominal surgery	135
Bowel / anastomotic remodeling stiffness	Follow-up stiffness by strain elastography or SWE	No universally accepted cutoff; increasing stiffness may suggest fibrotic remodeling or stricture risk (T)	Ultrasound strain elastography; SWE	Long-term follow-up of Crohn's strictures or postoperative anastomotic remodeling	20, 56, 91
Fluid shear stress	*τ* = *μ* (*∂u*/*∂y*)	Physiological-like shear ranges around 0.2-0.5 Pa in intestinal epithelial *in vitro* systems; not a direct bedside threshold (E)	Organ-on-chip; microfluidics; CFD simulation	Mechanistic modeling of epithelial barrier function and mechanobiology	49, 50

Note: Thresholds are classified as (C) clinically implemented, (T) translational, and (E) experimental. Quantitative values are modality-, organ-, and endpoint-dependent. Abbreviations: LS, liver stiffness; SWE, shear-wave elastography; MRE, magnetic resonance elastography; SWV, shear-wave velocity; POPF, postoperative pancreatic fistula; DI, distensibility index; EndoFLIP, endoscopic functional lumen imaging probe; ICG, indocyanine green; PVP, portal venous pressure; IAP, intra-abdominal pressure; IAH, intra-abdominal hypertension; ACS, abdominal compartment syndrome; CFD, computational fluid dynamics.

**Table 2:** Modulation of the mechanical microenvironment in digestive surgery across the perioperative continuum

Stage	Key mechanical parameters	Assessment / monitoring tools	Representative thresholds or examples	Typical clinical decisions / interventions	Maturity	References
Preoperative	Liver stiffness; fibrosis burden	MRE; 2D-SWE	LS < 8.6 kPa suggests lower-risk hepatectomy candidacy; LS > 12-15 kPa suggests advanced fibrosis/high-risk liver reserve	Adjust resection extent; consider staged surgery or intensified liver protection	C/T	21, 67, 68, 86
Preoperative	Pancreatic stiffness / gland texture	SWE; ultrasound elastography	Low pancreatic stiffness (e.g., SWV < 1.3 m/s) associated with higher POPF risk	Modify anastomotic strategy; consider stent placement, drain planning, or intensified fistula prevention	T	19, 46, 47
Preoperative	Tumor or stricture mechanical phenotype	SWE; MRE	Decrease in CRLM stiffness during chemotherapy may indicate response; higher bowel stiffness may suggest fibrotic rather than inflammatory phenotype	Refine timing of surgery; help distinguish surgery from medical therapy in fibrostenotic disease	T	20, 62, 90
Preoperative	Patient-specific tissue mechanics in simulation models	CT/MRI-based geometry; elastography-informed FE modeling	Outputs include predicted stress concentration, deformation, or remnant load	Optimize resection plane, anastomotic site, or reconstruction strategy *in silico*	T/E	22, 57, 94, 97, 101
Intraoperative	Lumen distensibility	EndoFLIP	Low DI indicates an overly tight wrap; selected studies suggest target DI ranges of about 2-4 mm^2^/mmHg or > 3.5 mm^2^/mmHg at 40 mL	Adjust wrap tightness or reconstruction before completion	C/T	40, 53, 107
Intraoperative	Anastomotic perfusion / conduit perfusion	Quantitative ICG fluorescence imaging	Delayed fluorescence or weak intensity suggests impaired perfusion	Revise transection line; select a better-perfused anastomotic site; consider diversion or reinforcement	C/T	59, 109, 110, 158
Intraoperative	Portal inflow and remnant liver hemodynamic load	Intraoperative portal pressure measurement	PVP ≥ 20 mmHg after resection or clamping suggests high risk in selected studies	Modify or stage hepatectomy; consider portal pressure modulation	T	88
Intraoperative	Tissue force transmission during suturing	Mechanical sensors; force-sensing concepts; slipknot-gauged systems	No universally accepted clinical threshold; device-assisted force control aims to reduce excessive traction	Improve suture force consistency; reduce cut-through risk; support robotic/assisted force control	T/E	38, 116, 117
Postoperative	Intra-abdominal pressure	Continuous bladder pressure monitoring; sensor-based IAP monitoring	IAP ≥ 12 mmHg = IAH; IAP ≥ 20 mmHg = ACS	Decompression, fluid optimization, abdominal pressure management, escalation of surveillance	C	135, 140, 141, 146, 147
Postoperative	Early warning of leak, distension, or impaired recovery	Implantable pH/impedance sensors; wearable abdominal strain sensors; wireless motility monitoring	No standardized threshold; abnormal dynamic trends may indicate leak, distension, or ileus/obstruction	Trigger imaging, drainage, antibiotic treatment, nutritional adjustment, or reoperation assessment	T/E	140, 143-145, 147
Postoperative	Mechanical support and load redistribution	Bioresorbable scaffolds; reinforcement patches; abdominal support	Benefit inferred from improved tensile strength, load sharing, or reduced local stress in translational studies	Reinforce vulnerable anastomoses or closures; reduce peak stress during early healing	T/E	117, 130-133
Postoperative / rehabilitation	Functional loading, motility, and tissue remodeling	Early mobilization; enteral nutrition; follow-up elastography	Early mobilization and early enteral feeding support recovery; increasing follow-up stiffness may suggest fibrotic remodeling or stricture risk	Promote perfusion and motility; tailor rehabilitation; intensify surveillance or intervene for stricture-prone healing	C/T	20, 56, 91, 134, 135, 138, 139

Note: Technologies are classified by clinical maturity as (C) clinically implemented, (T) translational, and (E) experimental. Abbreviations: LS, liver stiffness; SWE, shear-wave elastography; MRE, magnetic resonance elastography; CRLM, colorectal liver metastases; FE, finite element; DI, distensibility index; EndoFLIP, endoscopic functional lumen imaging probe; ICG, indocyanine green; PVP, portal venous pressure; POPF, postoperative pancreatic fistula; IAP, intra-abdominal pressure; IAH, intra-abdominal hypertension; ACS, abdominal compartment syndrome.

## Abbreviations

2D-SWE: two-dimensional shear wave elastography
ACS: abdominal compartment syndrome
AI: artificial intelligence
ASBO: adhesive small-bowel obstruction
AUC: area under the curve
BP: burst pressure
bFGF: basic fibroblast growth factor
CFD: computational fluid dynamics
CRLM: colorectal liver metastases
CT: computed tomography
DI: distensibility index
ECM: extracellular matrix
EndoFLIP: endoscopic functional lumen imaging probe
ERAS: enhanced recovery after surgery
FAK: focal adhesion kinase
FE: finite element
FDA: U.S. Food and Drug Administration
HCC: hepatocellular carcinoma
IAH: intra-abdominal hypertension
IAP: intra-abdominal pressure
ICG: indocyanine green
LS: liver stiffness
MCI: mechanical compatibility index
MMP: matrix metalloproteinase
MRE: magnetic resonance elastography
MRI: magnetic resonance imaging
NO: nitric oxide
NPO: nil-by-mouth
PCL: poly-ε-caprolactone
POPF: postoperative pancreatic fistula
PSCs: pancreatic stellate cells
PVP: portal venous pressure
ROC: receiver operating characteristic
ROCK: Rho-associated kinase
SWE: shear-wave elastography
SWV: shear-wave velocity
TGF-β: transforming growth factor-beta
TRPV4: transient receptor potential vanilloid 4
VAC: vacuum-assisted closure
VEGF: vascular endothelial growth factor
YAP: Yes-associated protein
TAZ: transcriptional co-activator with PDZ-binding motif
Piezo1: piezo type mechanosensitive ion channel component 1
